# Profiling of Stem/Progenitor Cell Regulatory Genes of the Synovial Joint by Genome-Wide RNA-Seq Analysis

**DOI:** 10.1155/2018/9327487

**Published:** 2018-06-26

**Authors:** Yue Zhou, Mo Chen, Christopher L. Ricupero, Ling He, Jiaqian Wu, Kenian Chen, Richard A. Friedman, Paolo Guarnieri, Zuolin Wang, Xuedong Zhou, Jeremy J. Mao

**Affiliations:** ^1^State Key Laboratory of Oral Diseases, West China Hospital of Stomatology, West China School of Stomatology, Sichuan University, Chengdu 610041, China; ^2^Center for Craniofacial Regeneration, Columbia University Medical Center, 630 W. 168 St., PH7 East CDM, New York, NY 10032, USA; ^3^Shanghai Engineering Research Center of Tooth Restoration and Regeneration, School and Hospital of Stomatology, Tongji University, Shanghai 200072, China; ^4^Columbia University College of Dental Medicine, 630 W. 168 St., PH7E, New York, NY 10032, USA; ^5^Guanghua School of Stomatology, Hospital of Stomatology, Sun Yat-sen University, 56 Lingyuanxi Road, Guangzhou 510055, China; ^6^Vivian L. Smith Department of Neurosurgery, Center for Stem Cell and Regenerative Medicine, University of Texas Medical School at Houston, Houston, TX 77030, USA; ^7^Biomedical Informatics Shared Resource, Herbert Irving Comprehensive Cancer Center, Columbia University, New York, NY 10032, USA; ^8^Department of Systems Biology, Columbia University Medical Center, New York, NY 10032, USA

## Abstract

Synovial joints suffer from arthritis and trauma that may be severely debilitative. Despite robust investigations in the roles of individual genes in synovial joint development and arthritis, little is known about global profiles of genes that regulate stem/progenitor cells of a synovial joint. The temporomandibular joint is a poorly understood synovial arthrosis with few clinical treatment options. Here, we isolated the articular and mature zones of the mandibular condyle by laser capture microdissection, performed genome-wide profiling, and analyzed molecular signaling pathways relevant to stem/progenitor cell functions. A total of 804 genes were differentially expressed between the articular and mature zones. Pathway analyses revealed 29 enriched signaling pathways, including the PI3K-Akt, Wnt, and Toll-like receptor signaling pathways that may regulate stem/progenitor cell homeostasis and differentiation into the chondrocyte lineage. Upstream regulator analyses further predicted potential upstream key regulators such as Xbp1, Nupr1, and Hif1a, and associated underlying mechanism networks were described. Among the multiple candidates of growth and transcriptional factors that may regulate stem/progenitor cells, we immunolocalized Sox9, Ihh, Frzb, Dkk1, Lgr5, and TGF*β*3 in the articular and mature zones. These findings provide a comprehensive genetic mapping of growth and transcriptional genes in the articular and mature zones of a synovial joint condyle. Differentially expressed genes may play crucial roles in the regulation of stem/progenitor cells in development, homeostasis, and tissue regeneration.

## 1. Introduction

Synovial joint diseases are a substantial burden to society, including arthritis, trauma, and congenital anomalies. Individual growth and transcriptional factors have been studied primarily to understand joint development and arthritis [1, 2]. However, little is known regarding the genes that regulate stem/progenitor cells in homeostasis or pathological conditions of postnatal synovial joints. The temporomandibular joint is a complex synovial arthrosis with the mandibular condyle serving more than articular cartilage. During development, the mandibular condyle is both articular cartilage and an underlying growth plate [3, 4]. The mandibular condyle forms from a subset of neural crest derived mesenchymal stem cells that differentiate and secrete a matrix rich in type II collagen and proteoglycans [5, 6]. Despite the presence of fibrocartilage in the mandibular disk which is functionally equivalent to the knee meniscus, the mandibular condyle shares numerous characteristics of a synovial joint and serves as a pivotal site for bone growth [7, 8].

The mandibular condyle is divided into three distinctive zones in the sagittal plane: articular zone (az), mature zone (mz), and hypertrophic zone (hz) [9]. The articular zone is the most superficial layer and immersed in the synovial cavity. Cells in the articular zone are densely packed during development, with some of the cells active in lubricin synthesis [10]. Some of the articular zone cells display stem/progenitor cell characteristics [11–13]. The mature zone lies underneath the articular zone and primarily consists of chondrocytes with abundant extracellular matrix such as aggrecan and type II collagen [14]. In the mature zone, cell morphology gradually changes from flattened to spherical shape. In the hypertrophic zone, chondrocytes undergo terminal differentiation and become hypertrophic with enlarged size and deposition of type X collagen [15], followed by mineralization of cartilaginous matrix and newly formed bone [16]. Thus, stem/progenitor cell differentiation from the superficial, articular zone towards mature and hypertrophic zones provides a powerful model for understanding growth and transcriptional genes that regulate the behavior of stem/progenitor cells. Despite previous demonstrations of isolated growth and transcriptional factors in arthritis and development [17, 18], a comprehensive global analysis of gene expression profiles of the temporomandibular joint condyle is unavailable. Here, we profiled global gene expression of different zones of the mandibular condyle by laser capture microdissection and RNA-Seq analysis with a specific focus on growth and transcriptional factors that may regulate stem/progenitor cell behavior.

## 2. Materials and Methods

### 2.1. Tissue Preparation

Animal use protocol was approved by Columbia University Institutional Animal Care and Use Committee (IACUC). Mandibular condyles of postnatal 7-day CD-1 mice (Charles River Laboratory, Stone Ridge, NY) were surgically removed after inhalational anesthesia and immediately embedded in optimum cutting temperature compound (Leica Biosystems), frozen on dry ice, and stored at −80°C until sectioning. Frozen sections (10-*μ*m thickness) were prepared using a cryostat at −20°C and were mounted on precooled PEN membrane-covered slides (Zeiss). Slides were stored at −80°C until further processing.

### 2.2. Tissue Staining

Slides were removed from −80°C and thawed at room temperature. After rethawing, slides were fixed in 70% ethanol for 2 min and dipped 5-6 times in RNase-free distilled water to remove optimum cutting temperature compound. Slides were then stained with Mayer's Hematoxylin (Sigma-Aldrich) for 1 min, washed in DEPC-treated water for 1 min, stained with Eosin Y (Sigma-Aldrich) for 10 s, and dehydrated subsequently using ethanol series (70%, 96%, 100%). All aqueous reagents were prepared with diethylpyrocarbonate- (DEPC-) treated or RNase-free water.

### 2.3. Laser Capture Microdissection (LCM)

LCM was performed using the PALM Microbeam system (Zeiss). The articular zone, mature zone, and hypertrophic zone were readily identified on the basis of cell morphology under 10×microscope objective. A focused laser beam was activated to dissect the zone of interest into Adhesive Cap tube caps (Zeiss). A total of 25-30 sections from each zone were microdissected and pooled to create single samples. A total of three independent biological replicates were tested.

### 2.4. RNA Extraction and RNA Quality Assay

The collected cell samples were immediately lysed in 50-*μ*l Arcturus PicoPure RNA extraction buffer at 42°C for 30 min. Total RNA was extracted from cell lysates using Arcturus PicoPure RNA Isolation Kit (Applied Biosystems). To evaluate RNA quality, the RNA 6000 Pico kit and Bio-Analyzer 2100 (Agilent Biotechnologies) were used. RNA Integrity Number (RIN) ranging from 10 (intact) to 1 (totally degraded) was analyzed and only high-quality RNA samples (RIN value>6) were selected for RNA-sequencing.

### 2.5. RNA-Sequencing and Data Analysis

RNA samples were subjected to RNA-sequencing at the University of Rochester Genomics Research Center. Briefly, total RNAs from each sample were polyA selected and single-end sequencing libraries were constructed using TruSeq RNA Sample Prep Kit (Illumina). The samples were then sequenced using the Illumina HiSeq sequencer. The RNA-Seq reads were cleaned according to a rigorous preprocessing workflow (Trimmomatic-0.32) and mapped to the Mus musculus genome (version mm10) with SHRiMP2.2.3. Differential expressed genes were identified using DESeq, with <0.05 false discovery rate (FDR). Fragments Per Kilobase of exon model per Million Mapped fragments (FPKM) were estimated for each gene in each sample. Hierarchical clustering was also performed on log_2_FPKM values with Cluster 3.0. Only genes with FDR<0.05 and ≥0.80 standard deviation were included. Results were displayed as a heatmap with JavaTreeview. Pathway analysis was performed to investigate potential interactions of differently regulated genes. Ingenuity Pathway Analysis (IPA) was used to generate the network of connections between modulated genes, using knowledge-based topology. Upstream Regulator and Mechanistic Regulator Algorithms were used to infer regulatory networks responsible for the observed differential expression.

### 2.6. qRT-PCR Validation

The cDNA was synthesized with the iScript cDNA synthesis kit (Bio-Rad). SYBR green-based RT-PCR reactions were performed using the ViiA™ 7 system (Life technologies) according to the manufacturer's protocol. Primers used for the RT-PCR are listed in Supplementary Table 4 with all reactions in triplicate to detect the cycle thresholds value. Gene expressions normalised to Gapdh were analyzed by the 2^−ΔΔCt^ method with statistically significance determined using Student's* t*-tests.

### 2.7. Immunohistochemistry

Mandible condyle samples were fixed in 10% formalin, decalcified in EDTA, and then embedded in paraffin. Tissue sections (5*μ*m thickness) were prepared and immunohistochemistry was performed with primary antibodies (Abcam) and HRP-DAB System Staining Kit (R&D) per manufacturer protocols. Primary antibodies included polyclonal rabbit anti-mouse Sox9, Ihh, Frzb, Dkk1, Lgr5, and Tgf*β*3.

## 3. Results

**Figure 1:**
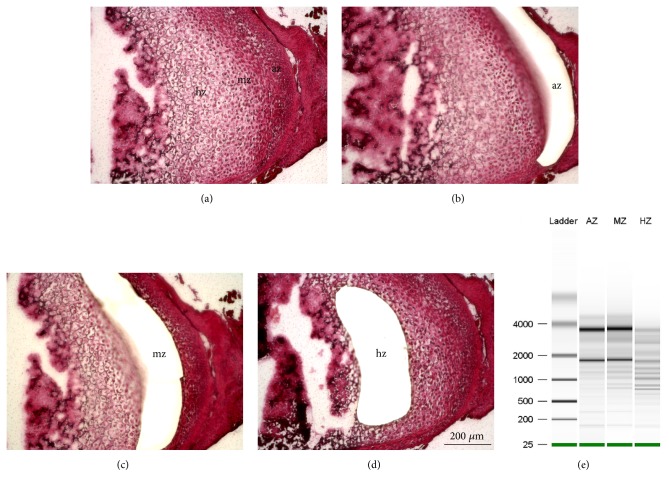
**Laser capture microdissection (LCM) of synovial joint condyle.** (a) HE-staining showed the location and morphology of articular zone (az), mature zone (mz), and hypertrophic zone (hz) of mandibular condyle of postnatal day 7 (P7) mouse. (b-d) Laser capture of az, mz and hz, respectively. (e) Representative bioanalyzer electropherograms showing the position of 18S and 28S rRNA peaks, indicating RNA quality from each cell zones. Scale bar=200 *μ*m.

Mandibular condyles of postnatal 7-day-old mice were frozen-sectioned with articular, mature and hypertrophic zones identified under a dissection microscope (Figure 1(a)). Cells from the articular zone (az), mature zone (mz), and hypertrophic zone (hz) were isolated by laser capture microscopy (LCM) (Figures 1(b)–1(d)). A total of ~10-40 ng of RNA samples from each zone in three biological replicates were subjected to RNA quality analysis. RIN numbers (RNA Integrity Number) (Figure 1(e) and Supplementary Table 1) were acceptable for articular and mature zone samples (RIN 6.0-7.8) but were consistently unacceptable for hypertrophic zone samples (RIN 2.7-5.3), likely attributable to apoptosis of hypertrophic chondrocytes. Therefore, only the articular and mature zone samples were deemed sufficient for RNA-sequencing.

**Figure 2:**
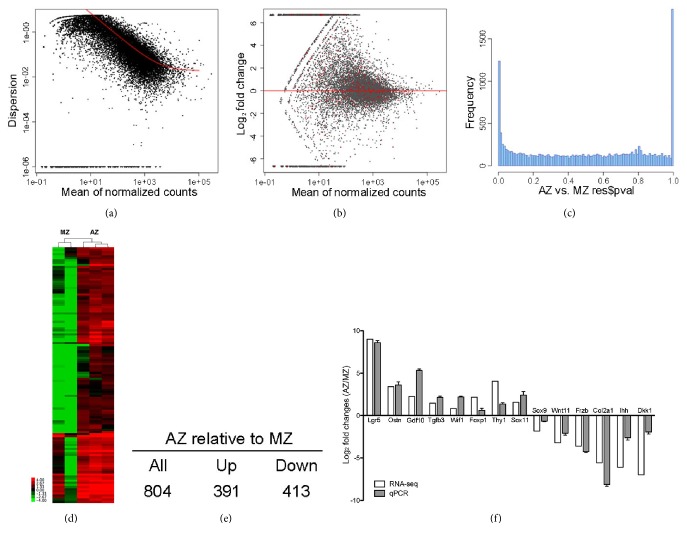
**RNA-sequencing analysis of differential gene expression between articular zone and mature zone of the synovial joint condyle.** (a) Empirical (black dots) and fitted (red lines) dispersion values plotted against the mean of the normalised count. (b) Plot of normalised mean versus log2 fold change for the contrast AZ versus MZ. (c) Histogram of P values from gene-by-gene statistical tests for differential expression. (d) Heatmap with hierarchical clustering of gene expression in articular zone (az) and mature zone (mz). (e) Summary of differential gene expression by the comparison of articular zone to mature zone. (f) Validation of RNA-Seq results by RT-qPCR.

Total sequencing reads representing RNA transcripts were generated and were mapped to the NCBI mouse reference genome (Supplementary Table 2). DESeq was used to estimate the statistical significance of differential gene expression profiles between zones (Figures 2(a)–2(c)). RNA comparison between articular and mature zones revealed distinctive gene expression profiles, as illustrated by the heatmap with an FDR (false discovery rate) <0.05 (Figure 2(d)). A total of 804 genes showed significantly differential expression between the articular and mature zones (az versus mz): 391 upregulated and 413 downregulated (Figure 2(e) and Supplementary Table 3).

To verify the sequencing transcripts, 14 differentially expressed genes of relevance to the regulation of chondrogenic differentiation were selected for qRT-PCR. Ihh and Col2a1 are known to be expressed and play critical roles in the growth and differentiation of condylar cartilage [19, 20]. Sox9 is also required for TMJ morphogenesis [21], raising the possibility that a member of the Sox family Sox11 might similarly play roles in TMJ maintenance. Ostn is secreted by osteoblast and other mesenchymal cells to modulate bone growth and chondrocyte proliferation through binding to Natriuretic Peptide Clearance Receptor [22, 23]. Foxp1 and Thy1 are of particular interest since Foxp1 has previously demonstrated a critical function in fate choice of mesenchymal stem cell differentiation [24], and Thy1 was identified as a stem cell marker [25]. The functional significance of Wnt and TGF*β* signaling in TMJ development has been implicated by the spatiotemporal expression patterns of Wnt signaling pathway [26, 27], upregulation of *β*-catenin [27], or conditional inactivation of TGF*β*2 [28], raising the possibility that some members of the Wnt and BMP signaling pathways, including Lgr5, Wif1, Wnt11, Frzb, Dkk1, Gdf10, and TGf*β*3, may be involved in TMJ formation and homeostasis. Our qRT-PCR results confirmed upregulation of Lgr5, Ostn, Gdf10, Tgf*β*3, Wif1, Foxp1, Thy1, and Sox11 and downregulation of Sox9, Wnt11, Frzb, Col2a1, Ihh, and Dkk1 in the articular zone relative to the mature zone (Figure 2(f)).

**Figure 3:**
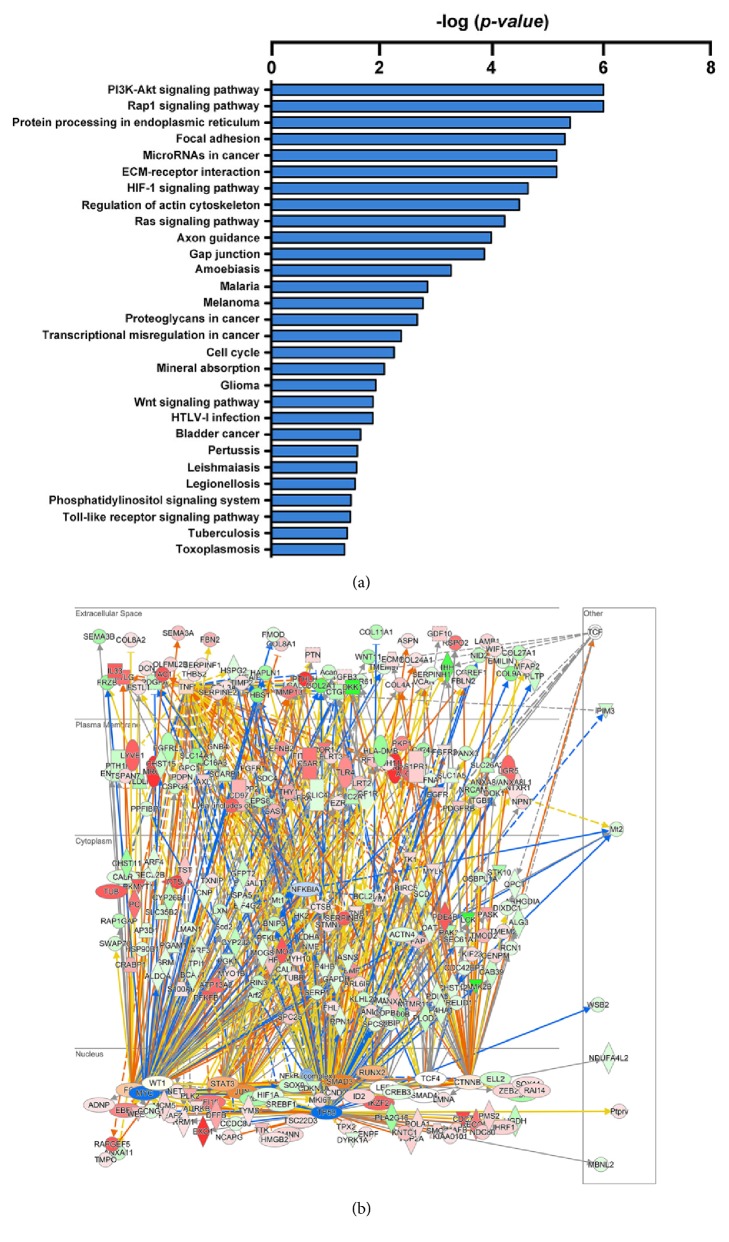
**Enriched signaling pathways and Ingenuity Pathway Analysis (IPA).** (a) The top 29 significantly enriched signaling pathways in the Ingenuity Pathway database indicate differences in biological processes between articular and mature zones. (b) Network regulating observed differential expression inferred by the Ingenuity Upstream Regulator and Mechanistic Network Algorithms.

Differentially expressed genes between the articular and mature zones suggest different molecular mechanisms to regulate cells in corresponding zones in homeostasis. Accordingly, we performed overrepresentation analysis against the Ingenuity Pathway database and detected 29 differentially enriched signaling pathways with statistical significance (Figure 3(a) and Supplementary Table 5). In addition, to infer the identity of upstream regulatory molecules and associated mechanisms to provide biological insight into the observed gene expression changes, upstream regulator and mechanistic network analysis were performed, based on a large-scale causal network derived from the Ingenuity Knowledge Base. The upstream regulator analysis identified 19 activated and 38 inhibited upstream regulators, including target molecules, shown in Supplementary Table 6. The gene regulatory networks that illustrated the causal regulations were depicted in Figure 3(b).

**Figure 4:**
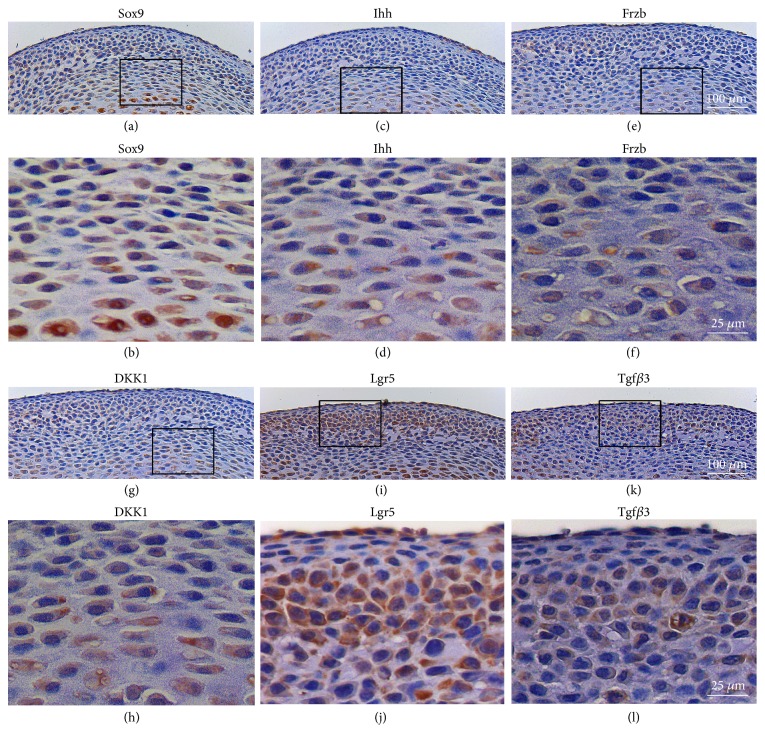
**Immunohistochemistry of chondrogenesis related genes. **(a-b) Sox9 was strongly expressed in both articular zone and mature zone. (c-d) Ihh was modestly positive in the articular zone, but intensely expressed in the mature zone. (e-h) Frzb and Dkk1 were detected in mature zone at a relative low level. (i, j) Lgr5 expression was primarily in the articular zone, but modest in the mature zone. (k, l) Tgf*β*3 showed a similar expression pattern to Lgr5. Scale bar = 100 *μ*m (a, c, e, g, i, k). Scale bar=25 *μ*m (b, d, f, h, j, l).

Among multiple candidates genes that may regulate stem/progenitor cells, we immunolocalized Sox9, Ihh, Frzb, Dkk1, Lgr5, and TGF*β*3 in the articular and mature zones (Figure 4). Sox9 was strongly expressed in both articular zone and mature zone, whereas Ihh was modestly positive in the articular zone, but intensely expressed in the mature zone. Frzb and Dkk1 were detected in mature zone at a relatively low level. Contrastingly, the greatest Lgr5 expression was found in the articular zone, whereas Tgf*β*3 showed a similar but modest expression pattern to Lgr5. These findings confirm the RNA-sequencing transcriptional profile and provide a comprehensive expression mapping of growth and transcriptional genes in the articular and mature zones of a synovial joint condyle.

## 4. Discussion

The present genomic profiling dataset from the articular and mature zones of the synovial joint condyle may have implications in the understanding of development, homeostasis, and pathological conditions including arthritis. In several synovial joints, putative stem/progenitor cells are found to reside primarily in the superficial or articular zone of the articular cartilage [13, 29, 30]. The superficial zone has unique structural and mechanical properties that differ from the mature zone [31]. Although several growth and transcriptional factors have been previously reported to be associated with chondrogenesis [17–19], few genetic factors regulating chondrogenesis of stem/progenitor cells have been identified, highlighting the importance of further investigation into the functional interplay of multiple genes.

The present pathway enrichment analysis of differentially regulated genes suggested that 29 signaling pathways were significantly relevant to TMJ chondrogenic development. The importance of the PI3K/Akt pathway in the regulation of survival, proliferation, apoptosis, and differentiation of mesenchymal stem/progenitor cells has been demonstrated [32]. Our finding of a robustly enhanced PI3K/Akt pathway in the articular zone of condylar cartilage indicates that the manipulation of the PI3K/Akt pathway is a putative modulator of TMJ homeostasis and cartilage regeneration. The Wnt signaling pathway is known to be a critical regulator of TMJ chondrogenesis and osteoarthritis [13, 26, 33–36], although the mechanisms by which this pathway exerts its effects are still not fully understood. Here, we identified that Wnt-related genes Dkk1, Frzb, and Wnt11 were downregulated, while Lgr5, Fzd1, Sfrp4, Wif1, Tcf4, and Tcf19 were upregulated in the articular zone relative to the mature zone of the condylar cartilage, suggesting the dynamic roles of the Wnt signaling pathway in cartilage development and providing evidence to uncover potential key regulators in the specification of TMJ fibrochondrocyte differentiation. The pathway analysis also implicated additional signaling pathways including the Rap1, Hif-1, Ras, and Toll-like receptor signaling pathways as enriched in TMJ formation and homeostasis. Further functional investigations in each of the enriched signaling pathways would be required.

Upstream regulator analysis determines likely upstream regulators that can explain the observed gene expression changes in the dataset, which can help illuminate the biological activities occurring in the cells or tissues being studied. It has recently been used to identify TGF-*β*, TNF, and MYC as important upstream regulators in the differentiation transition of chondrocytes [37]. In the present study, the top predictions for upstream regulators of the observed gene expression profiles were Xbp1, Nupr1, and Hif1a. Xbp1 is a transcription factor critical for cell fate determination in response to endoplasmic reticulum stress [38]. Nupr1 was originally identified as p8, a member of the family of HMG-I/Y transcription factors induced in response to various cellular stressors [39]. Hif1a, a critical mediator of the cellular response to hypoxia, plays a significant role in chondrocyte proliferation, maturation, and differentiation [40]. These observations reemphasize the importance of stressors and hypoxic microenvironment challenged by the chondrocytes. Our genome-wide analysis of gene expression further reveals the potential underlying mechanism as well as insight into unexplored functional interplay of candidate key regulators and target genes for further study.

TMJ arthritis remains a poorly understood cluster of diseases, and due to poor understanding of its causes, clinical management is palliative. The presenting findings provide clues to further understand TMJ pathological conditions including arthritis. For example, TGF*β* signaling is crucial in the regulation of chondrocyte hypertrophy both in arthritis progression and in cartilage regeneration. Inhibition of TGF*β* signaling in chondrocytes results in a progressive osteoarthritis-like phenotype [41, 42]. Our data reveal that members of the TGF*β* superfamily, including Tgf*β*3 and Gdf10 (Bmp3), are differentially expressed between articular and mature zones of the mandibular condyle, indicating TGF*β*'s dynamic roles in gene expression during chondrocyte differentiation that may be of relevance to the progression of TMJ disease.

## 5. Conclusions

The present profiling data provide a comprehensive genetic mapping of growth and transcriptional factors in the synovial joint condyle. The identified gene expression profiles and signaling pathways analysis provide a baseline for additional investigations of insights into stem/progenitor cells in the development, homeostasis, and pathological conditions of synovial joint.

## Data Availability

RNA-sequencing (including raw and processed datasets) are available through the NCBI GEO database, accession code: GSE113116.

## Supplementary Materials

Supplementary Materials Supplementary Table 1. RNA quality from articular zone (az), mature zone (mz), and hypertrophic zone (hz) in three biological replicates was determined by RNA Integrity Number (RIN). Supplementary Table 2. Total sequencing reads representing RNA transcripts were generated and were mapped to the NCBI mouse reference genome. Supplementary Table 3. The 804 differentially expressed genes between the articular and mature zones in TMJ condylar cartilage were listed with fold change and statistical significance. Supplementary Table 4. Primer sequences were used for the qRT-PCR. Supplementary Table 5. The top 29 significantly enriched signaling pathways with statistical significance value were listed. Supplementary Table 6. IPA upstream regulator analysis identified 19 activated and 38 inhibited upstream regulators, including target molecules, based on the 804 differentially expressed genes between the articular and mature zones of TMJ condylar cartilage.
