# Supplementary material for: Profiling of Stem/Progenitor Cell Regulatory Genes of the Synovial Joint by Genome-Wide RNA-Seq Analysis
Source: Biomed Res Int. 2018 Jun 26;2018:9327487. doi: 10.1155/2018/9327487 (PMC6038687; doi:10.1155/2018/9327487)
Supplement: Supplementary Materials — Supplementary Table 1. RNA quality from articular zone (az), mature zone (mz), and hypertrophic zone (hz) in three biological replicates was determined by RNA Integrity Number (RIN). Supplementary Table 2. Total sequencing reads representing RNA transcripts were generated and were mapped to the NCBI mouse reference genome. Supplementary Table 3. The 804 differentially expressed genes between the articular and mature zones in TMJ condylar cartilage were listed with fold change and statistical significance. Supplementary Table 4. Primer sequences were used for the qRT-PCR. Supplementary Table 5. The top 29 significantly enriched signaling pathways with statistical significance value were listed. Supplementary Table 6. IPA upstream regulator analysis identified 19 activated and 38 inhibited upstream regulators, including target molecules, based on the 804 differentially expressed genes between the articular and mature zones of TMJ condylar cartilage. [file 9327487.f1.docx]

**Supplementary Table1. RNA evaluation**

| Samples | RNA area | RNA ratio (28s/18s) | RNA integrity number (RIN) | RNA harvested (ng) |
| --- | --- | --- | --- | --- |
| AZ-1 | 561.5 | 1.3 | 7.1 | 12 |
| AZ-2 | 226.7 | 1.7 | 7.7 | 40 |
| AZ-3 | 2101.7 | 1.8 | 7.8 | 38 |
| MZ-1 | 1106 | 2.1 | 6 | 22 |
| MZ-2 | 838.8 | 1.3 | 6.8 | 18 |
| MZ-3 | 2165.9 | 2.3 | 7.7 | 38 |
| HZ-1 | 1025 | 0.9 | 2.7 | 20 |
| HZ-2 | 405.6 | 0.6 | 3.2 | 9 |
| HZ-3 | 450.7 | 0.6 | 5.3 | 8 |

**Supplementary Table2. Sequencing and mapping of biological replicates**

| Samples | #Total reads (M) | # Mapped reads | % Mapped |
| --- | --- | --- | --- |
| AZ-1 | 28.45 | 24117514 | 84.75 |
| AZ-2 | 24.91 | 22594848 | 90.70 |
| AZ-3 | 31.03 | 27997427 | 90.21 |
| MZ-1 | 24.36 | 22299082 | 91.51 |
| MZ-2 | 26.65 | 24013829 | 90.20 |
| MZ-3 | 11362 | 97419 | 0.84 |

**Supplementary Table3. The 804 differentially expressed genes with statistical significance**

| **Gene** | **Foldchange** | **Log2Foldchange** | **pval** | **padj** |
| --- | --- | --- | --- | --- |
| \| Sgms2 \| \| --- \| \| Loxl4 \| \| Car12 \| \| Chst11 \| \| Smpd3 \| \| Frzb \| \| Susd5 \| \| C1qtnf3 \| \| Sort1 \| \| Ugdh \| \| Klhl13 \| \| Cd24a \| \| Scin \| \| Slc26a2 \| \| Car8 \| \| Cdc7 \| \| Igsf10 \| \| Eps8l2 \| \| Nid2 \| \| Mfsd7c \| \| Col11a2 \| \| Zcchc5 \| \| Tubb2b \| \| Fam102a \| \| Ptprd \| \| Ptn \| \| Pak3 \| \| Ctgf \| \| Ehd3 \| \| Bnip3 \| \| Sema3b \| \| Ywhaq \| \| Rnf157 \| \| Acan \| \| Anxa11 \| \| Scd1 \| \| Fam167a \| \| Col9a1 \| \| Top2a \| \| Plod2 \| \| Gale \| \| Sfrp4 \| \| Thbs1 \| \| Gprc5c \| \| Tubb2a \| \| Scarb1 \| \| Acot7 \| \| Pgam1 \| \| Fap \| \| Slc6a15 \| \| Zfp14 \| \| Egfr \| \| Hapln1 \| \| Fgfrl1 \| \| Ppm1e \| \| Lcp2 \| \| Sdc4 \| \| Pgm2 \| \| Fndc3b \| \| Klhl30 \| \| Ldlrad3 \| \| Sox9 \| \| Sec61a1 \| \| Olfml2b \| \| Prkg2 \| \| Vldlr \| \| Mt1 \| \| Gfpt2 \| \| Mtus2 \| \| 1810055G02Rik \| \| Fam111a \| \| Mest \| \| Papss2 \| \| Gpc1 \| \| Srpx \| \| Lims2 \| \| Lgals3 \| \| Fam20b \| \| P4ha2 \| \| Fgfr3 \| \| 2210020M01Rik \| \| Aspn \| \| Id2 \| \| Cspg4 \| \| Procr \| \| 4933409K07Rik \| \| Asxl3 \| \| Fmo1 \| \| Ugp2 \| \| D0H4S114 \| \| Ihh \| \| Fry \| \| Fam129b \| \| Pth1r \| \| Col2a1 \| \| Zfp41 \| \| Mki67 \| \| Ptprv \| \| Plod3 \| \| Timp2 \| \| Dcn \| \| Slc16a3 \| \| Aldh1l2 \| \| Sema3a \| \| Slc8a3 \| \| Slc35b2 \| \| Rab3il1 \| \| Fam180a \| \| Bcat1 \| \| Fv1 \| \| Aldoa \| \| Fgfr2 \| \| Ndrg4 \| \| Tmem97 \| \| Thbs2 \| \| Golph3 \| \| Pacsin2 \| \| Tacc1 \| \| Tmem45a \| \| Cab39 \| \| H47 \| \| Scd2 \| \| Ldha \| \| Arf2 \| \| Ednra \| \| Kcns3 \| \| Frem1 \| \| Pthlh \| \| Tac1 \| \| Clint1 \| \| Plxna2 \| \| Luzp2 \| \| Islr \| \| Impad1 \| \| Matn1 \| \| Pdgfrb \| \| Bysl \| \| Kdelr3 \| \| Gjc3 \| \| Tpi1 \| \| Prelp \| \| Fzd1 \| \| Hlf \| \| Ssr3 \| \| Emilin1 \| \| Tmem100 \| \| Matn3 \| \| Sec24a \| \| Serpinb9 \| \| Serp1 \| \| Lamb1 \| \| Surf4 \| \| Ckap4 \| \| Rapgef5 \| \| Zfhx4 \| \| Ccdc47 \| \| Cyr61 \| \| Serpinf1 \| \| Pkm2 \| \| Pde4b \| \| Pask \| \| Rai14 \| \| Pgk1 \| \| Fam198a \| \| AI597468 \| \| Gapdh \| \| Taf13 \| \| Spc25 \| \| Mtmr11 \| \| Ndufa4l2 \| \| Epb4.1l2 \| \| Slc13a5 \| \| Npnt \| \| Col9a3 \| \| Lrrc59 \| \| Mylk \| \| Cdh11 \| \| Alg9 \| \| Glcci1 \| \| Hmcn1 \| \| Vdr \| \| Kctd12b \| \| Gdf10 \| \| Srprb \| \| Atp8a1 \| \| Pfkl \| \| Pkp1 \| \| Efna5 \| \| Stmn1 \| \| Scamp2 \| \| Kera \| \| Bcl2l1 \| \| Gimap6 \| \| Uhrf1 \| \| Col8a2 \| \| Tcf4 \| \| Wee1 \| \| Pla2g16 \| \| Chsy1 \| \| Adamts2 \| \| Col9a2 \| \| Ebf1 \| \| Rhbdl2 \| \| Gys1 \| \| 6230409E13Rik \| \| Swap70 \| \| 2010106G01Rik \| \| Txnip \| \| Sidt2 \| \| Frk \| \| Itgb5 \| \| Lig1 \| \| Tmem170b \| \| Polm \| \| Panx3 \| \| Pdgfc \| \| Polq \| \| Chst15 \| \| Wnt11 \| \| Arf4 \| \| Cdkn1a \| \| Smc2 \| \| Aldoart1 \| \| Timeless \| \| Tbl2 \| \| Ly6c1 \| \| Fam89a \| \| Lin7a \| \| Col11a1 \| \| Dffb \| \| Zcchc24 \| \| Sec23a \| \| Ifitm3 \| \| A830007P12Rik \| \| Tspan9 \| \| Arhgap19 \| \| Serpinh1 \| \| Ano1 \| \| Wdr1 \| \| Gimap4 \| \| Lhfp \| \| Yipf6 \| \| Fam20a \| \| Srgap1 \| \| Lxn \| \| Vcan \| \| Sec22b \| \| Klhl21 \| \| Exo1 \| \| Mef2c \| \| Tmem66 \| \| Slc2a1 \| \| Cd200 \| \| Magel2 \| \| Cul7 \| \| Slc39a14 \| \| Tshz1 \| \| D6Mm5e \| \| Fhl1 \| \| Akirin1 \| \| Gab2 \| \| Mafb \| \| Fkbp11 \| \| Msi2 \| \| Fads3 \| \| Actn4 \| \| Kif18a \| \| Col3a1 \| \| Lect1 \| \| Mastl \| \| Ntan1 \| \| Gmds \| \| Trim2 \| \| Fkbp10 \| \| Dock11 \| \| Ikzf2 \| \| Anln \| \| Rnf2 \| \| Slc35d1 \| \| P4hb \| \| Igsf3 \| \| Gm13212 \| \| Dock8 \| \| Pltp \| \| A330049M08Rik \| \| Lclat1 \| \| Casc5 \| \| 2210403K04Rik \| \| Plekhg4 \| \| Zeb2 \| \| Slc39a13 \| \| Twf1 \| \| Srcrb4d \| \| Spon2 \| \| Plcd1 \| \| Gm3893 \| \| Scara3 \| \| Csgalnact1 \| \| Rpn1 \| \| Lman1 \| \| Adamts3 \| \| Zfp105 \| \| Adam22 \| \| Pdgfra \| \| Cpe \| \| Zfp106 \| \| Incenp \| \| Syne1 \| \| Tubb5 \| \| Rab38 \| \| Cdc42ep3 \| \| Trpv4 \| \| Fbln7 \| \| Col8a1 \| \| Tgfb3 \| \| Plk2 \| \| Galk1 \| \| Efnb2 \| \| Pdgfa \| \| Serpine2 \| \| Wwp2 \| \| Ctps2 \| \| Camk2b \| \| Ttk \| \| Gpr1 \| \| Fstl1 \| \| Tram1 \| \| Axl \| \| Mxra8 \| \| Hspa5 \| \| Yipf5 \| \| 3110079O15Rik \| \| Thy1 \| \| P4ha1 \| \| Nsun2 \| \| Cenpf \| \| Alg3 \| \| Bcl11b \| \| Cd97 \| \| Phldb1 \| \| Ly6a \| \| Col4a1 \| \| Cpxm2 \| \| Col24a1 \| \| Stk38l \| \| Kctd12 \| \| Nt5e \| \| Aox1 \| \| Il33 \| \| Tspan7 \| \| Slc39a7 \| \| Fam64a \| \| Sgol2 \| \| Osbpl6 \| \| Itgbl1 \| \| Srp72 \| \| Hells \| \| Nme1 \| \| Tk1 \| \| Gjc1 \| \| Atp1b3 \| \| Cenpe \| \| Deb1 \| \| Birc5 \| \| Ell2 \| \| Etv1 \| \| Comp \| \| Cyp26b1 \| \| Tst \| \| Ak4 \| \| Chst12 \| \| Stk10 \| \| Pard3b \| \| Sobp \| \| Ckap2l \| \| Prelid1 \| \| Atp8b2 \| \| Il13ra1 \| \| Recql4 \| \| Olfm1 \| \| Kif23 \| \| Esco2 \| \| Ganab \| \| Cyp2j6 \| \| Pcx \| \| Glb1l2 \| \| Golph3l \| \| Dixdc1 \| \| Calr \| \| Zmpste24 \| \| Lgr5 \| \| Ostn \| \| Nrcam \| \| Eif1a \| \| D17Wsu104e \| \| Dusp27 \| \| Cox4i2 \| \| Pdia5 \| \| Osbpl1a \| \| Fbxo7 \| \| 5031410I06Rik \| \| Gm973 \| \| Arap1 \| \| Dpt \| \| Slc14a1 \| \| Evc \| \| A930017M01Rik \| \| Hck \| \| Emcn \| \| Shisa4 \| \| 1110017F19Rik \| \| Tmem119 \| \| Mt2 \| \| Gm10471 \| \| Lck \| \| Crabp1 \| \| Pms2 \| \| Mcm5 \| \| Mbnl2 \| \| Pdpn \| \| Tmem2 \| \| Cdca2 \| \| Fam162a \| \| Zfp27 \| \| BC025920 \| \| Cgref1 \| \| 1600002H07Rik \| \| Zfp420 \| \| Lyve1 \| \| Lpar4 \| \| Lbh \| \| Ift81 \| \| Srm \| \| Adamts10 \| \| Wscd2 \| \| Tmpo \| \| Cdv3 \| \| Ccdc18 \| \| Ctsb \| \| Smarca2 \| \| Pim3 \| \| Rpa1 \| \| Pf4 \| \| Ppa1 \| \| Xbp1 \| \| Slc30a9 \| \| Sra1 \| \| Lepre1 \| \| Cdca5 \| \| Bmf \| \| Gprasp1 \| \| Dyrk1a \| \| Appbp2 \| \| Cdc42se1 \| \| Mbnl1 \| \| Rrm1 \| \| Tpx2 \| \| Ccdc50 \| \| Ctla2a \| \| Ptprk \| \| Glt25d2 \| \| Sorcs1 \| \| Tlr4 \| \| Enah \| \| Hip1r \| \| Gnb4 \| \| Ost4 \| \| Plxna4 \| \| Fmod \| \| Eif3b \| \| Plod1 \| \| Chtf18 \| \| Wif1 \| \| 2200002K05Rik \| \| Synj1 \| \| Slc2a10 \| \| Igf1r \| \| Fkbp14 \| \| AW551984 \| \| Unc5c \| \| Pola1 \| \| Tmem53 \| \| Cercam \| \| Ncapg \| \| Stat5b \| \| S100a6 \| \| Pde5a \| \| Hspg2 \| \| Plekhg5 \| \| Asns \| \| Stt3a \| \| Pgf \| \| Rnf144a \| \| D11Wsu99e \| \| Mfap4 \| \| Anxa3 \| \| Ppap2c \| \| Pla2r1 \| \| Htatsf1 \| \| Spns1 \| \| Fam120a \| \| Adamtsl1 \| \| Cdr2l \| \| Gars \| \| Tmem20 \| \| Aldoart2 \| \| B4galt4 \| \| Cenpl \| \| Pcolce2 \| \| Dqx1 \| \| Mmp13 \| \| Slc50a1 \| \| Oat \| \| Kank1 \| \| Scml2 \| \| Pcyt1b \| \| Ndc80 \| \| Gas1 \| \| Snta1 \| \| Itga9 \| \| Atg3 \| \| Sult5a1 \| \| Dkk1 \| \| Ppfibp1 \| \| H2afz \| \| Mfap2 \| \| Btbd3 \| \| Grhpr \| \| Cenpm \| \| Nfix \| \| Kntc1 \| \| Aurkb \| \| 1500015O10Rik \| \| Cgnl1 \| \| Ssr2 \| \| Ddah2 \| \| Tnfaip2 \| \| Fam13c \| \| Gmnn \| \| Cep110 \| \| Olfml1 \| \| Mcfd2 \| \| Srebf1 \| \| Wrb \| \| Txnl1 \| \| Ska1 \| \| Mthfr \| \| Rac2 \| \| Zfp169 \| \| Lonrf3 \| \| Bod1l \| \| Sox11 \| \| Qpct \| \| BC021891 \| \| Chpf2 \| \| Pbx1 \| \| Zfp811 \| \| Enpp2 \| \| Ptprg \| \| Ccdc80 \| \| Capn5 \| \| Mmab \| \| Cops7a \| \| Sar1b \| \| Efna1 \| \| Slc35b4 \| \| Lrrc1 \| \| Asf1a \| \| Plscr3 \| \| Flrt2 \| \| Ube4b \| \| Rftn1 \| \| B4galt1 \| \| Il11ra1 \| \| Plxdc2 \| \| Ube2l6 \| \| S100b \| \| Ebf4 \| \| Yif1a \| \| Rbm12b \| \| Pdzd11 \| \| Clic4 \| \| Zfp385b \| \| Entpd7 \| \| Leprel4 \| \| Tacc3 \| \| Sar1a \| \| Fgfr1 \| \| Mogs \| \| Cnp \| \| H2-DMb2 \| \| Hsp90b1 \| \| Ror1 \| \| Kitl \| \| Fam168a \| \| Capn6 \| \| Mrc1 \| \| Stk32b \| \| Echs1 \| \| Gpr125 \| \| Col22a1 \| \| Zfp937 \| \| Creb3 \| \| Yipf1 \| \| Eif1ad \| \| Parp8 \| \| Rcn1 \| \| Kcnk1 \| \| Pfkfb1 \| \| Gorasp2 \| \| Zfp868 \| \| Slc25a1 \| \| Vim \| \| Hat1 \| \| 2900056M20Rik \| \| 4930422G04Rik \| \| Murc \| \| Atp13a2 \| \| Slc1a5 \| \| 2810417H13Rik \| \| Sec13 \| \| Ankrd44 \| \| Slc30a7 \| \| Epha7 \| \| Preb \| \| Arl6ip1 \| \| Marcks \| \| Slc25a45 \| \| Itpripl1 \| \| Csmd3 \| \| Krtdap \| \| Pola2 \| \| Zik1 \| \| Ppfibp2 \| \| Ptgfrn \| \| Spcs2 \| \| Vezf1 \| \| Cilp2 \| \| Slc35a2 \| \| Ptprb \| \| Prkdc \| \| Gcfc1 \| \| Zfand1 \| \| Foxp1 \| \| Clec3a \| \| Ikbip \| \| Efcab1 \| \| Zfp367 \| \| Fam123b \| \| Impdh1 \| \| Magt1 \| \| Plekha2 \| \| Eif5 \| \| Flrt3 \| \| Copb2 \| \| Papss1 \| \| Eif2c1 \| \| E130306D19Rik \| \| Tfg \| \| Tm9sf3 \| \| Slc38a3 \| \| Fbn2 \| \| Lmna \| \| Anxa8 \| \| Fam98a \| \| Gja4 \| \| Dok1 \| \| Txnl4b \| \| Ing5 \| \| Nip7 \| \| Stk39 \| \| Clspn \| \| Tcf19 \| \| Alcam \| \| Trp53inp2 \| \| Ppp4r1l-ps \| \| Fam81a \| \| Zfp791 \| \| Ssh2 \| \| Hmgb2 \| \| Myh10 \| \| Slc9a2 \| \| Extl1 \| \| Slc30a5 \| \| Antxr1 \| \| Arcn1 \| \| Kif21a \| \| Rab27b \| \| Wsb2 \| \| Nfia \| \| Msrb3 \| \| Net1 \| \| C1s \| \| Eps8 \| \| Nfatc4 \| \| Pole \| \| Tmem47 \| \| Ifi203 \| \| Ecm1 \| \| Trim47 \| \| Ctso \| \| Ykt6 \| \| Ccng1 \| \| Zfp85-rs1 \| \| Loxl3 \| \| Copb1 \| \| Lrrtm2 \| \| Nudt11 \| \| Gatad2a \| \| Ablim1 \| \| Plscr4 \| \| Ap3d1 \| \| Phtf2 \| \| 5031426D15Rik \| \| Zfp932 \| \| Ube2d3 \| \| Diap2 \| \| Zfp386 \| \| Trerf1 \| \| Dclk1 \| \| Fbln2 \| \| Ufm1 \| \| Cep72 \| \| Tub \| \| Adnp \| \| Srp54a \| \| Traf7 \| \| Dpp3 \| \| Myo5a \| \| Myo1b \| \| Pde4dip \| \| Phip \| \| Ttyh3 \| \| Mtss1l \| \| Pkmyt1 \| \| Tyms \| \| Ccnb1 \| \| Slc38a4 \| \| Arid4b \| \| Clec14a \| \| Ufsp2 \| \| Bicc1 \| \| Tsc22d3 \| \| Arhgdia \| \| C5ar1 \| \| Pdzrn4 \| \| Hif1a \| \| Tmbim1 \| \| Plekhg6 \| \| Hk2 \| \| 1110067D22Rik \| \| Txndc5 \| \| Galnt4 \| \| Rspo2 \| \| Nalcn \| \| Arf3 \| \| Ano10 \| \| Tmod2 \| \| Itpkc \| \| St8sia2 \| \| Lrrfip1 \| \| C2cd2 \| \| Abca9 \| \| Rin3 \| \| Nomo1 \| \| Ostc \| \| S1pr1 \| \| Slc6a8 \| \| Calu \| \| Ptplad1 \| \| Ncln \| \| Cd81 \| \| Aim1l \| \| 2700049A03Rik \| \| Cpm \| \| Mcc \| \| Fbxl4 \| \| Atg13 \| \| Camk1d \| \| Mak16 \| \| Fancg \| \| Rab1 \| \| Chac1 \| \| E330016A19Rik \| \| Ctss \| \| 5830417I10Rik \| \| Eif4g2 \| \| Nr2f2 \| \| Vash2 \| \| Lrrn3 \| \| Bnip3l \| \| Col27a1 \| \| Ezr \| \| Usp40 \| \| Copz1 \| \| Gpihbp1 \| \| Ube2f \| \| Cep97 \| \| Sox8 \| \| Uevld \| \| Ccnd2 \| \| Rap1gap \| \| Cope \| \| Spin4 \| | \| 0.070766486 \| \| --- \| \| 0.04916501 \| \| 0.091904708 \| \| 0.09903478 \| \| 0.097576059 \| \| 0.081915736 \| \| 0.138714749 \| \| 0.266216236 \| \| 0.156028132 \| \| 0.211070173 \| \| 0.134515535 \| \| 0.115577218 \| \| 0.013143234 \| \| 0.180952485 \| \| 0.129635654 \| \| 340.7456413 \| \| 4.966934698 \| \| 0.035866493 \| \| 0.204232771 \| \| 0.051666649 \| \| 0.064772163 \| \| 0.223362914 \| \| 0.241700848 \| \| 0.185371866 \| \| 5.391141398 \| \| 3.333846393 \| \| 0.137521435 \| \| 0.191032091 \| \| 0.219367646 \| \| 0.246490932 \| \| 0.115266769 \| \| 0.355911803 \| \| 235.6349409 \| \| 0.285027743 \| \| 0.235601663 \| \| 0.32620972 \| \| 6.621944863 \| \| 0.055960454 \| \| 4.313565544 \| \| 0.240120018 \| \| 0.17611566 \| \| 811.3230875 \| \| 0.053257444 \| \| 0.161486028 \| \| 0.260710462 \| \| 0.300747513 \| \| 0.221298671 \| \| 0.388929892 \| \| 6.395848 \| \| 0.183050731 \| \| Inf \| \| 7.009396019 \| \| 0.100870088 \| \| 0.172126046 \| \| 0.157184084 \| \| 201.9315233 \| \| 0.419483795 \| \| 0.274425359 \| \| 0.362084072 \| \| 0.037785576 \| \| 0.246137623 \| \| 0.281196093 \| \| 0.417807197 \| \| 4.830372547 \| \| 0.037270323 \| \| 0.142617197 \| \| 0.321833847 \| \| 0.269969691 \| \| 0.001569808 \| \| 0.364790766 \| \| 9.715059912 \| \| 2.481499052 \| \| 0.141037198 \| \| 0.432508605 \| \| 0.285124308 \| \| 0.079725428 \| \| 0.284359934 \| \| 0.151590054 \| \| 0.283824852 \| \| 0.252102583 \| \| 0.030684385 \| \| 2.661328474 \| \| 3.9727995 \| \| 0.268264594 \| \| 590.1476977 \| \| 19.70753768 \| \| 0.171259661 \| \| 209.4424178 \| \| 0.315647649 \| \| 2.949900622 \| \| 0.0146157 \| \| 0.100814336 \| \| 0.309582927 \| \| 0.28097535 \| \| 0.0211134 \| \| 48.16116872 \| \| 4.39061266 \| \| 2.755646886 \| \| 0.370414019 \| \| 2.522781679 \| \| 2.199905432 \| \| 0.219755198 \| \| 0.277388864 \| \| 7.9299523 \| \| 0.154786228 \| \| 0.369763573 \| \| 18.90470808 \| \| 0.415253022 \| \| 0.365821322 \| \| Inf \| \| 0.486569127 \| \| 2.382713519 \| \| 0.278118272 \| \| 0.440522568 \| \| 2.782799543 \| \| 0.363964184 \| \| 0.323756306 \| \| 0.373413581 \| \| 0.318175906 \| \| 0.257218576 \| \| 0.42805135 \| \| 0.48645753 \| \| 0.46651909 \| \| 0.313764129 \| \| 22.96460525 \| \| 113.0777189 \| \| Inf \| \| 708.7309547 \| \| Inf \| \| 0.361198573 \| \| 120.9960444 \| \| 0.017550601 \| \| 7.863406147 \| \| 0.383752533 \| \| 0 \| \| 6.50375205 \| \| 0.201591814 \| \| 0.465742577 \| \| 0.001218583 \| \| 0.486230141 \| \| 0.25309645 \| \| 5.341474787 \| \| Inf \| \| 0.48745522 \| \| 0.421011708 \| \| 3.392728626 \| \| 0.016258476 \| \| 0.309440398 \| \| Inf \| \| 0.417298389 \| \| 4.051378538 \| \| 0.475395099 \| \| 0.490698364 \| \| Inf \| \| 2.892166771 \| \| 0.362408655 \| \| 0.349547929 \| \| 2.163381265 \| \| 0.517095664 \| \| Inf \| \| 23.06074746 \| \| 3.779138833 \| \| 0.368565941 \| \| Inf \| \| 0.461555234 \| \| 0.459796818 \| \| 0.316623085 \| \| 6.035046912 \| \| 10.0781702 \| \| 0.433140783 \| \| 0.404914733 \| \| 0.14566445 \| \| 3.886781739 \| \| 0.031850718 \| \| 0.462845827 \| \| 3.50342751 \| \| 2.683698822 \| \| 0.201366086 \| \| 3.701591913 \| \| 2.915744601 \| \| 0.11737121 \| \| Inf \| \| 4.733926629 \| \| 0.422965877 \| \| 197.9241076 \| \| 0.347924507 \| \| 53.42448171 \| \| 3.002389698 \| \| 2.830610807 \| \| 0.386714645 \| \| 50.15235286 \| \| 0.268438065 \| \| Inf \| \| 4.140948213 \| \| 2.820078702 \| \| 2.423078666 \| \| 9.797595822 \| \| 0.101594197 \| \| 0.457947441 \| \| 40.54522519 \| \| 0.046433251 \| \| 54.31812814 \| \| 0.217529345 \| \| 0.301753807 \| \| 28.82746951 \| \| 0.307367418 \| \| 0.396050561 \| \| 2.721703313 \| \| 0.193726896 \| \| 9.637858571 \| \| 2.522108545 \| \| 4.304290533 \| \| 0.370393321 \| \| 23.22904817 \| \| 0.258503009 \| \| Inf \| \| 36.2781017 \| \| 0.282778369 \| \| 0.108223507 \| \| 0.536015621 \| \| 0.304629372 \| \| 3.917214476 \| \| 0.49846123 \| \| 6.560747595 \| \| 0.336997076 \| \| Inf \| \| 0.093166286 \| \| 27.92145328 \| \| 0.171491488 \| \| 17.80234362 \| \| 6.844272947 \| \| 0.442054277 \| \| 3.550764084 \| \| 61.17893734 \| \| 3.492861596 \| \| 39.28903645 \| \| 0.549386833 \| \| 7.244447479 \| \| 0.484036899 \| \| Inf \| \| 5.715411109 \| \| 0.313807255 \| \| 8.704310823 \| \| 0.264227574 \| \| 0.29117067 \| \| 2.302106817 \| \| 0.456908626 \| \| 0.288527061 \| \| 811.4643933 \| \| 0.41540659 \| \| 0.339880099 \| \| 0.187888043 \| \| 0.350318426 \| \| 0.050254269 \| \| 0.286179557 \| \| 0.260673165 \| \| 5.405032193 \| \| 0.045721796 \| \| 2.193927565 \| \| 0.376152565 \| \| 40.90665179 \| \| 4.297038641 \| \| 0.488756597 \| \| 0.309115686 \| \| 0.289075629 \| \| 0.471623191 \| \| 21.99140319 \| \| 1.777732585 \| \| 0.084018797 \| \| 17.34597435 \| \| 0.337571077 \| \| 0.304765709 \| \| 4.102378533 \| \| 0.544469693 \| \| 7.187669395 \| \| Inf \| \| 3.27516863 \| \| 6.027713072 \| \| 0.199414462 \| \| 0.565716799 \| \| 2.112117092 \| \| 82.63682274 \| \| 0.208847119 \| \| 0.281140869 \| \| 0.049613709 \| \| 0.361267593 \| \| 7.054607906 \| \| 0.341858838 \| \| 5.879217478 \| \| 4.102405472 \| \| 0.417454348 \| \| 0.502740752 \| \| 0.003258814 \| \| 5.021168928 \| \| 0.331893254 \| \| 32.67671804 \| \| 3.263174048 \| \| 0.157709948 \| \| 0.539492202 \| \| 0.520202904 \| \| 0.047609471 \| \| 120.2266549 \| \| 4.444056097 \| \| 4.948965091 \| \| 0.259981389 \| \| 0.440624278 \| \| 6.182682989 \| \| 2.784650824 \| \| 1.934177086 \| \| Inf \| \| 0.458674426 \| \| 0.166711926 \| \| 0.113700663 \| \| 5.653009266 \| \| 2.759770585 \| \| 16.22449102 \| \| 0.391875076 \| \| 41.22398711 \| \| 0.21826838 \| \| 1.846593702 \| \| 0.250473036 \| \| 5.658357151 \| \| 0.005382018 \| \| 9.36676852 \| \| 73.27119821 \| \| 2.206217882 \| \| 0.503338263 \| \| 4.484758971 \| \| 2.170112022 \| \| 0.579701515 \| \| 0.462136853 \| \| 0.031043127 \| \| 16.55253181 \| \| 0.379677202 \| \| 0.482060195 \| \| 2.592492674 \| \| 0.313756944 \| \| 6.58840424 \| \| 10.91978875 \| \| 0.381650896 \| \| 20.64431259 \| \| 6.716916072 \| \| 14.87230119 \| \| 2.387239415 \| \| 0.416821136 \| \| 6.95756726 \| \| 0.480299377 \| \| Inf \| \| Inf \| \| 3.947070824 \| \| 0.54593519 \| \| 4.433557098 \| \| 4.311437284 \| \| 10.70242952 \| \| 293.3894767 \| \| 0.513858042 \| \| 7.45100025 \| \| 0.553355491 \| \| 3.807847176 \| \| 3.862710669 \| \| 0.341040322 \| \| 2.560197991 \| \| 0.231247921 \| \| 2.686227655 \| \| 0.304156296 \| \| Inf \| \| 0.015872299 \| \| 0.202554908 \| \| 7.001562618 \| \| 0.302274015 \| \| 0.456916277 \| \| 0.130224643 \| \| 5.875486026 \| \| 0.19482803 \| \| 3.286437958 \| \| 0.509402728 \| \| 0.455560858 \| \| 109.3535322 \| \| 86.17020818 \| \| 0.238686695 \| \| 3.132110532 \| \| 7.700662816 \| \| 0.51664127 \| \| 0.203969102 \| \| 98.89369768 \| \| Inf \| \| 12.04610981 \| \| 0.414969515 \| \| 0.583154773 \| \| 0.40180619 \| \| 158.849284 \| \| 10.59437358 \| \| 0.106648257 \| \| 0.514598251 \| \| 0.515154248 \| \| 0.013830487 \| \| 0.374914615 \| \| 0.409503224 \| \| 0.369915542 \| \| 0.237553966 \| \| 15.2202978 \| \| 0.071372109 \| \| 0.295618663 \| \| 3.927250411 \| \| 0.071630964 \| \| 4.053246691 \| \| 7.171649015 \| \| 0.285018266 \| \| Inf \| \| 0.226693485 \| \| 0.059726106 \| \| 0.521354607 \| \| 0.363811162 \| \| 26.42389272 \| \| 0 \| \| 15.2014723 \| \| 4.478721133 \| \| 3.874066391 \| \| 0.556242884 \| \| 5.364015006 \| \| 2.195453133 \| \| 4.275989159 \| \| 0.519310453 \| \| 9.195421833 \| \| 133.1940144 \| \| 0.175608752 \| \| 13.04806846 \| \| 59.28791415 \| \| Inf \| \| 0.376589342 \| \| 0.122107581 \| \| 6.071400925 \| \| 0.464070977 \| \| 3.390267657 \| \| 0.157910029 \| \| 2.263383489 \| \| 0.561172866 \| \| 12.96777439 \| \| 1.794115401 \| \| 2.486752029 \| \| 0.313941903 \| \| 2.618776717 \| \| 134.9344194 \| \| 0.466095607 \| \| 0.50014818 \| \| 0.367349513 \| \| 0.542156796 \| \| 0.552084091 \| \| 6.40460674 \| \| 38.23384367 \| \| 5.011869649 \| \| 0.481259578 \| \| 0.398145666 \| \| 0.495561187 \| \| 0.286095479 \| \| 2.250870698 \| \| 2.952244342 \| \| 2.165114455 \| \| Inf \| \| 8.24243517 \| \| 0.248456446 \| \| Inf \| \| 38.1032275 \| \| 0.386443381 \| \| 0.04074744 \| \| 0.479760706 \| \| 0.403926636 \| \| 0.235393022 \| \| 0.556832398 \| \| 0.544579833 \| \| 0.477652718 \| \| 11.77461873 \| \| 1.780773101 \| \| 40.44273309 \| \| 5.528696428 \| \| 0.388784713 \| \| 0.524029724 \| \| 0.530897595 \| \| 10.46842036 \| \| 0.115488064 \| \| 3.610857325 \| \| 250.6409688 \| \| 0.485266121 \| \| 3.152133197 \| \| 4.083437783 \| \| 1.84836822 \| \| 19.65725711 \| \| 0.436858935 \| \| 11.6930536 \| \| 0.515616224 \| \| 0.593540394 \| \| Inf \| \| 0.471339237 \| \| 0.359111053 \| \| 10.28509562 \| \| 8.145403404 \| \| 0.232315807 \| \| 146.4842975 \| \| 0.407360171 \| \| 0.370670139 \| \| 0.552568001 \| \| 5.720756522 \| \| 0.470493551 \| \| 0.535503344 \| \| 0.028084906 \| \| 0.508336931 \| \| 220.6215471 \| \| 3.15661957 \| \| 0.049635707 \| \| 0.211258921 \| \| 73.8522943 \| \| 2.840033744 \| \| 0.560347434 \| \| 0.233856086 \| \| 7.609677717 \| \| Inf \| \| 5.423300613 \| \| 11.28435009 \| \| 0.241856892 \| \| 4.889895343 \| \| 0.384317886 \| \| Inf \| \| 0.007929625 \| \| 0.362018123 \| \| 1.693848212 \| \| 2.658525181 \| \| 0.192451467 \| \| 0.353544407 \| \| 3.92909946 \| \| 2.092940664 \| \| 8.449838402 \| \| 2.814876344 \| \| 1.780270658 \| \| 5.266125547 \| \| 0.619791771 \| \| 7.507522903 \| \| 0.348734321 \| \| Inf \| \| 4.139184229 \| \| 2.802507808 \| \| 4.004228558 \| \| 0.464347302 \| \| 0.439460544 \| \| 0.304837535 \| \| 0.470995385 \| \| 6.226892857 \| \| 7.301924845 \| \| Inf \| \| 22.3624373 \| \| 64.55807431 \| \| 2.313085996 \| \| 2.956039894 \| \| 0.47932971 \| \| 0.119481465 \| \| 0.427619019 \| \| 11.8528264 \| \| 17.5007714 \| \| 2.58706415 \| \| 6.588632565 \| \| 0.544629296 \| \| 115.2414819 \| \| 4.339650678 \| \| 0.513623195 \| \| 0.505208547 \| \| 0.462654378 \| \| 0.428494735 \| \| 0.243568152 \| \| 8.454769475 \| \| 3.792157788 \| \| 7.612028856 \| \| 0.458448002 \| \| 7.641464783 \| \| 1.942917241 \| \| 5.515091789 \| \| 10.1629205 \| \| 5.502594322 \| \| 0.064021856 \| \| Inf \| \| 0.483028388 \| \| 9.066765259 \| \| 0.468846627 \| \| 0.566641505 \| \| 0.052332106 \| \| 0.450362519 \| \| 0.503486691 \| \| 2.112080254 \| \| 0.610501736 \| \| 2.00604334 \| \| 0.460183314 \| \| 0.453748903 \| \| 0.092962381 \| \| 0.635516096 \| \| 81.83536045 \| \| Inf \| \| 2.289796229 \| \| 0.641653777 \| \| 900.9207609 \| \| 27.86397201 \| \| 2.214361042 \| \| 0.461066255 \| \| 8.574091105 \| \| 12.00196898 \| \| 0.54306162 \| \| 0.481339677 \| \| 0.466532349 \| \| 2.582553155 \| \| 0.578110513 \| \| 0.392023954 \| \| 199.7208076 \| \| 0.586006304 \| \| 2.242900431 \| \| 0.339204212 \| \| 1.568643239 \| \| 3.011482426 \| \| 13.98826288 \| \| 3.95719873 \| \| 0.154952804 \| \| Inf \| \| 0.47354293 \| \| 2.597158302 \| \| 0.603496723 \| \| 2.481098923 \| \| 0.450478065 \| \| Inf \| \| 0.259753217 \| \| 2.365994685 \| \| 1.75984637 \| \| 40.35221082 \| \| 7.720012095 \| \| 35.301432 \| \| 12.00513145 \| \| 16.60217165 \| \| 66.65338711 \| \| 2.576462815 \| \| 1.817131663 \| \| 0.5617499 \| \| 2.519688861 \| \| 35.30614352 \| \| 0.411938636 \| \| 296.105769 \| \| 4.745645461 \| \| 2.46749327 \| \| 5.601620054 \| \| 4.480534351 \| \| 0.020573518 \| \| 0.530182563 \| \| 0.340231731 \| \| 2.3574381 \| \| 3.148003008 \| \| 0.384873579 \| \| 0.543491256 \| \| 5.238585061 \| \| 0.571317553 \| \| 3.559884655 \| \| 0.608065798 \| \| 0.541885849 \| \| 4.432905686 \| \| 8.256917352 \| \| 0.560786026 \| \| 0.567912754 \| \| 0.020197234 \| \| 13.06598681 \| \| 1.603456663 \| \| 0.372036424 \| \| 0.485679399 \| \| Inf \| \| 0.179032386 \| \| 9.218948535 \| \| 3.291959132 \| \| 0.543030657 \| \| 0.371800811 \| \| 5.795777237 \| \| 2.803958838 \| \| 190.011448 \| \| 0.253535501 \| \| 5.737481796 \| \| 0.1088218 \| \| Inf \| \| 32.74399512 \| \| 1.874820812 \| \| 1.915222946 \| \| 63.66384475 \| \| 0.128892634 \| \| 0.383494248 \| \| 1.843662918 \| \| 0.609806722 \| \| 0.377330913 \| \| 3.004734067 \| \| 0.395198285 \| \| 6.455577993 \| \| 0.436028695 \| \| 2.103784883 \| \| 17.24560539 \| \| 0.225112404 \| \| 3.271047854 \| \| 2.988851748 \| \| 4.934459862 \| \| Inf \| \| 2.99170582 \| \| 0.307302399 \| \| 69.52548973 \| \| 0.514844501 \| \| 0.432679016 \| \| 16.14339684 \| \| 0.109078098 \| \| 0.616477033 \| \| 111.2633853 \| \| Inf \| \| 0.504339036 \| \| 15.67043046 \| \| 9.193051561 \| \| 0.518740109 \| \| 0.429500118 \| \| Inf \| \| 5.560900905 \| \| 0.597625258 \| \| 4.951012157 \| \| 3.463725359 \| \| 36.00698178 \| \| 2.003146347 \| \| 3.524099066 \| \| 0.52618407 \| \| 28.15752344 \| \| 182.7682064 \| \| 2.039772552 \| \| 0.581664646 \| \| 2.215998728 \| \| 0.423565066 \| \| 0.573492599 \| \| 1.963815762 \| \| 0.356467739 \| \| 2.164036649 \| \| 3.997750554 \| \| 0.360380396 \| \| 199.1147304 \| \| 2.185298077 \| \| 2.017790091 \| \| 0.472983504 \| \| 2.380081586 \| \| Inf \| \| 0.567563812 \| \| 2.68455479 \| \| 3.104248396 \| \| 0.618110112 \| \| 78.62214227 \| \| 2.82697518 \| \| 0.555710088 \| \| 0.528851619 \| \| 0 \| \| 0.351124917 \| \| 0.459851457 \| \| 0.628208279 \| \| 9.691043656 \| \| Inf \| \| 0.149498041 \| \| 0.483787668 \| \| 0.245881946 \| \| 12.16049049 \| \| 13.7310949 \| \| Inf \| \| 0.401256127 \| \| 4.966732375 \| \| 7.951620952 \| \| 0.264655256 \| \| 0.507974555 \| \| 0.627335378 \| \| 4.686420837 \| \| 0.461826168 \| \| 0.667836824 \| \| 0.468262043 \| \| 0.471227735 \| \| 1.549424248 \| \| 0.151378315 \| \| 3.199208861 \| \| 28.40019487 \| \| 4.07649839 \| \| 5.351056375 \| \| 0.369537757 \| \| 3.119231242 \| \| 0.444073549 \| \| 7.952280946 \| \| 0.621053289 \| \| 0.262885333 \| \| 18.73614166 \| \| Inf \| \| 3.094669746 \| \| 0.663700472 \| \| 3.739658047 \| \| 5.534585385 \| \| 12.82575859 \| \| 0.569517932 \| \| 0.201959742 \| \| 2.758823057 \| \| 4.591721397 \| \| 0.615297269 \| \| 509.4657532 \| \| 0.354707055 \| \| 7.30808107 \| \| 0.109924391 \| \| 6.431845737 \| \| 1.583241358 \| \| 0.188753621 \| \| 0.63212376 \| \| 37.60777509 \| | \| -3.820789908 \| \| --- \| \| -4.346224256 \| \| -3.443717425 \| \| -3.335920919 \| \| -3.357328976 \| \| -3.609715571 \| \| -2.849806903 \| \| -1.909329535 \| \| -2.680121925 \| \| -2.244205371 \| \| -2.894155295 \| \| -3.113071048 \| \| -6.249535854 \| \| -2.466317176 \| \| -2.947465539 \| \| 8.412551393 \| \| 2.312355779 \| \| -4.801219486 \| \| -2.29171372 \| \| -4.274622887 \| \| -3.948482264 \| \| -2.162538426 \| \| -2.048705557 \| \| -2.431505793 \| \| 2.430590749 \| \| 1.737187634 \| \| -2.862271588 \| \| -2.388113085 \| \| -2.188577331 \| \| -2.020393519 \| \| -3.116951442 \| \| -1.490408318 \| \| 7.880409674 \| \| -1.810825746 \| \| -2.085578372 \| \| -1.616128327 \| \| 2.727254998 \| \| -4.159448529 \| \| 2.108880878 \| \| -2.058172414 \| \| -2.505404901 \| \| 9.664132733 \| \| -4.230872995 \| \| -2.630518751 \| \| -1.939479616 \| \| -1.733375289 \| \| -2.175933306 \| \| -1.362417975 \| \| 2.677135653 \| \| -2.449684558 \| \| Inf \| \| 2.809290136 \| \| -3.309429667 \| \| -2.538462677 \| \| -2.669472953 \| \| 7.657722336 \| \| -1.253313017 \| \| -1.86551429 \| \| -1.46560338 \| \| -4.726020587 \| \| -2.022462896 \| \| -1.830351543 \| \| -1.259090752 \| \| 2.272134463 \| \| -4.745828867 \| \| -2.809780142 \| \| -1.635612032 \| \| -1.889130645 \| \| -9.315195779 \| \| -1.454858883 \| \| 3.280222893 \| \| 1.311211904 \| \| -2.825852377 \| \| -1.209199258 \| \| -1.810337052 \| \| -3.648816255 \| \| -1.814209889 \| \| -2.721752991 \| \| -1.816927176 \| \| -1.987917193 \| \| -5.026351529 \| \| 1.412146585 \| \| 1.990155985 \| \| -1.898271437 \| \| 9.204932256 \| \| 4.300675628 \| \| -2.545742721 \| \| 7.710409846 \| \| -1.663613091 \| \| 1.560666353 \| \| -6.096337301 \| \| -3.310227281 \| \| -1.691602183 \| \| -1.831484527 \| \| -5.565697248 \| \| 5.589798497 \| \| 2.134422265 \| \| 1.46239103 \| \| -1.432789393 \| \| 1.335015361 \| \| 1.137441507 \| \| -2.186030805 \| \| -1.850018225 \| \| 2.987312188 \| \| -2.691650978 \| \| -1.435324989 \| \| 4.240673667 \| \| -1.267937428 \| \| -1.45078893 \| \| Inf \| \| -1.039283311 \| \| 1.252605502 \| \| -1.846229564 \| \| -1.182712165 \| \| 1.476536989 \| \| -1.458131608 \| \| -1.627019802 \| \| -1.421153694 \| \| -1.652103504 \| \| -1.958933258 \| \| -1.22414422 \| \| -1.039614237 \| \| -1.099991977 \| \| -1.672247668 \| \| 4.52134008 \| \| 6.821170875 \| \| Inf \| \| 9.469094252 \| \| Inf \| \| -1.469135901 \| \| 6.918816074 \| \| -5.832335779 \| \| 2.975154372 \| \| -1.381751822 \| \| -Inf \| \| 2.701272257 \| \| -2.310491036 \| \| -1.102395318 \| \| -9.680579245 \| \| -1.040288767 \| \| -1.982240826 \| \| 2.417238127 \| \| Inf \| \| -1.036658404 \| \| -1.248067739 \| \| 1.762446038 \| \| -5.942664187 \| \| -1.692266538 \| \| Inf \| \| -1.260848744 \| \| 2.018412889 \| \| -1.072801064 \| \| -1.027091635 \| \| Inf \| \| 1.532150745 \| \| -1.464310686 \| \| -1.516437808 \| \| 1.113287942 \| \| -0.951496889 \| \| Inf \| \| 4.52736737 \| \| 1.918057519 \| \| -1.440005337 \| \| Inf \| \| -1.11542479 \| \| -1.120931612 \| \| -1.659161649 \| \| 2.593364985 \| \| 3.333161821 \| \| -1.207092075 \| \| -1.304309958 \| \| -2.779279267 \| \| 1.958576096 \| \| -4.972530317 \| \| -1.111396381 \| \| 1.808767045 \| \| 1.424222774 \| \| -2.312107368 \| \| 1.888145852 \| \| 1.543864355 \| \| -3.090849519 \| \| Inf \| \| 2.243037346 \| \| -1.241386816 \| \| 7.628803536 \| \| -1.523153791 \| \| 5.739429102 \| \| 1.586111245 \| \| 1.5011134 \| \| -1.370658696 \| \| 5.64824548 \| \| -1.897338831 \| \| Inf \| \| 2.04996116 \| \| 1.495735426 \| \| 1.276841243 \| \| 3.292427778 \| \| -3.299110093 \| \| -1.126746067 \| \| 5.34146012 \| \| -4.428697888 \| \| 5.763361858 \| \| -2.200718061 \| \| -1.728556122 \| \| 4.849372297 \| \| -1.701963851 \| \| -1.336243476 \| \| 1.44450981 \| \| -2.36790383 \| \| 3.268712631 \| \| 1.334630367 \| \| 2.105775461 \| \| -1.432870011 \| \| 4.537858134 \| \| -1.951747021 \| \| Inf \| \| 5.181027062 \| \| -1.822256327 \| \| -3.207914203 \| \| -0.899653049 \| \| -1.714873044 \| \| 1.969828121 \| \| -1.004446797 \| \| 2.713860219 \| \| -1.569192023 \| \| Inf \| \| -3.4240482 \| \| 4.803302129 \| \| -2.543791126 \| \| 4.153995275 \| \| 2.774897295 \| \| -1.177704576 \| \| 1.82812951 \| \| 5.934963143 \| \| 1.804409477 \| \| 5.296054882 \| \| -0.864105761 \| \| 2.856875662 \| \| -1.046811063 \| \| Inf \| \| 2.514857275 \| \| -1.672049388 \| \| 3.121730075 \| \| -1.920147067 \| \| -1.780063056 \| \| 1.202954776 \| \| -1.130022415 \| \| -1.793221458 \| \| 9.664383981 \| \| -1.267403993 \| \| -1.556902206 \| \| -2.412054835 \| \| -1.513261221 \| \| -4.314610035 \| \| -1.805007476 \| \| -1.939686023 \| \| 2.434303211 \| \| -4.450974119 \| \| 1.133515894 \| \| -1.410610166 \| \| 5.354263552 \| \| 2.10334275 \| \| -1.032811918 \| \| -1.693781228 \| \| -1.79048111 \| \| -1.084293433 \| \| 4.458867755 \| \| 0.830038323 \| \| -3.573144069 \| \| 4.116528976 \| \| -1.566736793 \| \| -1.714227507 \| \| 2.036460618 \| \| -0.87707635 \| \| 2.845524052 \| \| Inf \| \| 1.711569189 \| \| 2.591610744 \| \| -2.326158051 \| \| -0.821848082 \| \| 1.078689817 \| \| 6.368712881 \| \| -2.259480855 \| \| -1.830634905 \| \| -4.333117363 \| \| -1.468860248 \| \| 2.8185659 \| \| -1.548527372 \| \| 2.555624145 \| \| 2.036470092 \| \| -1.260309658 \| \| -0.992113457 \| \| -8.261437252 \| \| 2.328023263 \| \| -1.591208788 \| \| 5.030191185 \| \| 1.706275938 \| \| -2.664654428 \| \| -0.890325988 \| \| -0.942853643 \| \| -4.392607603 \| \| 6.909612974 \| \| 2.151877028 \| \| 2.307126866 \| \| -1.943519743 \| \| -1.182379107 \| \| 2.628233035 \| \| 1.477496435 \| \| 0.951719888 \| \| Inf \| \| -1.124457625 \| \| -2.584570779 \| \| -3.136687427 \| \| 2.499019062 \| \| 1.464548343 \| \| 4.020101315 \| \| -1.351534278 \| \| 5.365412141 \| \| -2.19582495 \| \| 0.884866471 \| \| -1.997272791 \| \| 2.500383241 \| \| -7.537637186 \| \| 3.227551412 \| \| 6.195174303 \| \| 1.141575276 \| \| -0.990399822 \| \| 2.165030451 \| \| 1.117769517 \| \| -0.786617839 \| \| -1.113607952 \| \| -5.009582294 \| \| 4.048979998 \| \| -1.397154722 \| \| -1.052714788 \| \| 1.374339912 \| \| -1.672280705 \| \| 2.719929076 \| \| 3.448873041 \| \| -1.389674518 \| \| 4.367672476 \| \| 2.747799003 \| \| 3.894555988 \| \| 1.255343261 \| \| -1.26249966 \| \| 2.79858295 \| \| -1.057994158 \| \| Inf \| \| Inf \| \| 1.980782406 \| \| -0.873198403 \| \| 2.148464656 \| \| 2.108168894 \| \| 3.41986643 \| \| 8.196673315 \| \| -0.960558237 \| \| 2.897434111 \| \| -0.853721489 \| \| 1.928975579 \| \| 1.949613619 \| \| -1.55198577 \| \| 1.356255384 \| \| -2.112487697 \| \| 1.425581577 \| \| -1.717115228 \| \| Inf \| \| -5.977345093 \| \| -2.303615055 \| \| 2.807676941 \| \| -1.726071131 \| \| -1.129998257 \| \| -2.940925607 \| \| 2.554708198 \| \| -2.359726844 \| \| 1.71652475 \| \| -0.97312141 \| \| -1.134284299 \| \| 6.772856011 \| \| 6.429117264 \| \| -2.066809944 \| \| 1.647135126 \| \| 2.944982628 \| \| -0.952765202 \| \| -2.293577467 \| \| 6.627806678 \| \| Inf \| \| 3.59049541 \| \| -1.268922741 \| \| -0.778049261 \| \| -1.315428304 \| \| 7.31151478 \| \| 3.405226382 \| \| -3.229067705 \| \| -0.958481541 \| \| -0.956923626 \| \| -6.176004187 \| \| -1.415366029 \| \| -1.288053286 \| \| -1.434732179 \| \| -2.0736728 \| \| 3.927924682 \| \| -3.808495779 \| \| -1.75819074 \| \| 1.973519591 \| \| -3.803272833 \| \| 2.019077984 \| \| 2.842304884 \| \| -1.810873713 \| \| Inf \| \| -2.141185165 \| \| -4.065494524 \| \| -0.939663119 \| \| -1.45873829 \| \| 4.723771113 \| \| -Inf \| \| 3.926139154 \| \| 2.16308684 \| \| 1.953848678 \| \| -0.846213119 \| \| 2.423313273 \| \| 1.134518737 \| \| 2.096258195 \| \| -0.945330829 \| \| 3.200915759 \| \| 7.05738544 \| \| -2.509563346 \| \| 3.705764352 \| \| 5.889666136 \| \| Inf \| \| -1.408935926 \| \| -3.033775327 \| \| 2.602029445 \| \| -1.107582622 \| \| 1.761399177 \| \| -2.662825298 \| \| 1.178481044 \| \| -0.833482843 \| \| 3.696858991 \| \| 0.84327269 \| \| 1.314262654 \| \| -1.671430492 \| \| 1.388893058 \| \| 7.076114592 \| \| -1.10130218 \| \| -0.999572507 \| \| -1.444774732 \| \| -0.883217944 \| \| -0.857040067 \| \| 2.679109988 \| \| 5.256778337 \| \| 2.325348893 \| \| -1.055112841 \| \| -1.328631743 \| \| -1.012864896 \| \| -1.805431393 \| \| 1.170483183 \| \| 1.561812131 \| \| 1.114443292 \| \| Inf \| \| 3.043070635 \| \| -2.008935124 \| \| Inf \| \| 5.2518413 \| \| -1.371671039 \| \| -4.617146751 \| \| -1.059613094 \| \| -1.307834811 \| \| -2.086856544 \| \| -0.844684941 \| \| -0.876784537 \| \| -1.06596602 \| \| 3.557608441 \| \| 0.832503705 \| \| 5.337808592 \| \| 2.466939358 \| \| -1.362956601 \| \| -0.932279448 \| \| -0.91349449 \| \| 3.387971857 \| \| -3.114184337 \| \| 1.852341416 \| \| 7.969478441 \| \| -1.043151952 \| \| 1.656328499 \| \| 2.029784246 \| \| 0.88625219 \| \| 4.296990123 \| \| -1.194760596 \| \| 3.547579829 \| \| -0.955630435 \| \| -0.752581878 \| \| Inf \| \| -1.08516231 \| \| -1.477498035 \| \| 3.362483301 \| \| 3.025986151 \| \| -2.105840776 \| \| 7.194602212 \| \| -1.295623164 \| \| -1.431792199 \| \| -0.855776077 \| \| 2.516205944 \| \| -1.087753146 \| \| -0.90103251 \| \| -5.154061204 \| \| -0.976143048 \| \| 7.785429889 \| \| 1.6583804 \| \| -4.33247786 \| \| -2.242915832 \| \| 6.206570835 \| \| 1.505908071 \| \| -0.835606471 \| \| -2.09630712 \| \| 2.927835354 \| \| Inf \| \| 2.439171141 \| \| 3.496251425 \| \| -2.047774445 \| \| 2.289803588 \| \| -1.379627975 \| \| Inf \| \| -6.978531654 \| \| -1.465866172 \| \| 0.760304598 \| \| 1.410626131 \| \| -2.377433425 \| \| -1.500036657 \| \| 1.974198688 \| \| 1.065531411 \| \| 3.078923751 \| \| 1.493071547 \| \| 0.832096593 \| \| 2.396741916 \| \| -0.690144495 \| \| 2.908336971 \| \| -1.519799739 \| \| Inf \| \| 2.049346462 \| \| 1.486718392 \| \| 2.001524324 \| \| -1.106723841 \| \| -1.186194451 \| \| -1.713887539 \| \| -1.086215171 \| \| 2.638512456 \| \| 2.86827682 \| \| Inf \| \| 4.483005532 \| \| 6.01252564 \| \| 1.209818903 \| \| 1.56366574 \| \| -1.06090973 \| \| -3.065141261 \| \| -1.225602074 \| \| 3.567159217 \| \| 4.12934661 \| \| 1.371315828 \| \| 2.719979073 \| \| -0.876653508 \| \| 6.848516307 \| \| 2.117578917 \| \| -0.961217739 \| \| -0.98504905 \| \| -1.111993252 \| \| -1.222650618 \| \| -2.03760259 \| \| 3.079765419 \| \| 1.923018995 \| \| 2.928281031 \| \| -1.125169985 \| \| 2.933849213 \| \| 0.95822445 \| \| 2.463384897 \| \| 3.345243142 \| \| 2.46011197 \| \| -3.965291683 \| \| Inf \| \| -1.049820115 \| \| 3.180587934 \| \| -1.092812042 \| \| -0.819491816 \| \| -4.256159859 \| \| -1.150841328 \| \| -0.989974451 \| \| 1.078664655 \| \| -0.711932697 \| \| 1.004352775 \| \| -1.119719422 \| \| -1.14003394 \| \| -3.427209173 \| \| -0.65399943 \| \| 6.354652451 \| \| Inf \| \| 1.195219217 \| \| -0.640133035 \| \| 9.815256411 \| \| 4.800329024 \| \| 1.146890467 \| \| -1.116954016 \| \| 3.099983747 \| \| 3.585199201 \| \| -0.880812188 \| \| -1.054872744 \| \| -1.099950974 \| \| 1.368798043 \| \| -0.790582786 \| \| -1.350986283 \| \| 7.641840835 \| \| -0.771011909 \| \| 1.165365577 \| \| -1.559774011 \| \| 0.649517274 \| \| 1.59047384 \| \| 3.806144908 \| \| 1.984479519 \| \| -2.690099233 \| \| Inf \| \| -1.078432873 \| \| 1.376933952 \| \| -0.728582158 \| \| 1.310979258 \| \| -1.150471237 \| \| Inf \| \| -1.944786478 \| \| 1.242446833 \| \| 0.815449491 \| \| 5.334575813 \| \| 2.948603108 \| \| 5.141654803 \| \| 3.585579295 \| \| 4.053300061 \| \| 6.058606285 \| \| 1.365391771 \| \| 0.861662956 \| \| -0.832000133 \| \| 1.333245596 \| \| 5.14184734 \| \| -1.279498652 \| \| 8.209968788 \| \| 2.246604323 \| \| 1.30304615 \| \| 2.485844132 \| \| 2.163670799 \| \| -5.603067674 \| \| -0.915438873 \| \| -1.555410397 \| \| 1.23721989 \| \| 1.654436919 \| \| -1.37754346 \| \| -0.879671271 \| \| 2.389177193 \| \| -0.807635241 \| \| 1.831830497 \| \| -0.717700649 \| \| -0.883939123 \| \| 2.148252668 \| \| 3.045603265 \| \| -0.834477695 \| \| -0.816258783 \| \| -5.629698468 \| \| 3.707744183 \| \| 0.681185362 \| \| -1.426484222 \| \| -1.041923801 \| \| Inf \| \| -2.481707504 \| \| 3.204602214 \| \| 1.718946426 \| \| -0.880894448 \| \| -1.427398179 \| \| 2.535002145 \| \| 1.487465171 \| \| 7.569942532 \| \| -1.979740323 \| \| 2.520417671 \| \| -3.1999605 \| \| Inf \| \| 5.033158452 \| \| 0.906752715 \| \| 0.937512342 \| \| 5.992402381 \| \| -2.955758275 \| \| -1.382723157 \| \| 0.882574908 \| \| -0.713576041 \| \| -1.406097798 \| \| 1.587237311 \| \| -1.339351408 \| \| 2.690546271 \| \| -1.197505013 \| \| 1.072987193 \| \| 4.108156869 \| \| -2.151282543 \| \| 1.709752866 \| \| 1.579591339 \| \| 2.302892172 \| \| Inf \| \| 1.58096832 \| \| -1.702269063 \| \| 6.119470097 \| \| -0.957791335 \| \| -1.208630941 \| \| 4.012872272 \| \| -3.19656665 \| \| -0.697880948 \| \| 6.797835097 \| \| Inf \| \| -0.987534201 \| \| 3.969972905 \| \| 3.200543832 \| \| -0.946916173 \| \| -1.219269567 \| \| Inf \| \| 2.475318628 \| \| -0.742686972 \| \| 2.307723492 \| \| 1.792324542 \| \| 5.170204768 \| \| 1.002267826 \| \| 1.817254481 \| \| -0.926360524 \| \| 4.815448544 \| \| 7.513871317 \| \| 1.028408291 \| \| -0.781740476 \| \| 1.147957053 \| \| -1.239344488 \| \| -0.802153227 \| \| 0.973659587 \| \| -1.48815658 \| \| 1.113724932 \| \| 1.999188456 \| \| -1.472407561 \| \| 7.637456145 \| \| 1.127830078 \| \| 1.0127761 \| \| -1.080138226 \| \| 1.251011028 \| \| Inf \| \| -0.81714549 \| \| 1.42468285 \| \| 1.634244004 \| \| -0.694064227 \| \| 6.296863769 \| \| 1.499259217 \| \| -0.847595665 \| \| -0.919065096 \| \| -Inf \| \| -1.509943716 \| \| -1.120760183 \| \| -0.670685139 \| \| 3.276652042 \| \| Inf \| \| -2.741801512 \| \| -1.047554099 \| \| -2.023962285 \| \| 3.604129516 \| \| 3.779374763 \| \| Inf \| \| -1.317404674 \| \| 2.312297011 \| \| 2.991248986 \| \| -1.917813788 \| \| -0.977171863 \| \| -0.672691171 \| \| 2.228486513 \| \| -1.114578173 \| \| -0.582432451 \| \| -1.094611995 \| \| -1.085503641 \| \| 0.631732222 \| \| -2.723769542 \| \| 1.677715182 \| \| 4.827828924 \| \| 2.027330445 \| \| 2.419823728 \| \| -1.436206318 \| \| 1.64119051 \| \| -1.171129454 \| \| 2.991368727 \| \| -0.687211031 \| \| -1.927494443 \| \| 4.227751984 \| \| Inf \| \| 1.629785457 \| \| -0.591395795 \| \| 1.902906357 \| \| 2.468475244 \| \| 3.680972253 \| \| -0.812186826 \| \| -2.307860352 \| \| 1.464052929 \| \| 2.199035109 \| \| -0.700644506 \| \| 8.99284136 \| \| -1.495300071 \| \| 2.869492638 \| \| -3.185416554 \| \| 2.685232805 \| \| 0.662881204 \| \| -2.405423772 \| \| -0.661721051 \| \| 5.232959053 \| | \| 1.52E-47 \| \| --- \| \| 1.45E-40 \| \| 2.16E-34 \| \| 4.70E-33 \| \| 1.33E-29 \| \| 8.81E-28 \| \| 9.08E-28 \| \| 1.64E-25 \| \| 2.14E-23 \| \| 2.05E-22 \| \| 7.46E-22 \| \| 5.12E-21 \| \| 7.41E-21 \| \| 1.05E-20 \| \| 2.32E-20 \| \| 1.85E-19 \| \| 3.97E-19 \| \| 6.95E-19 \| \| 5.76E-18 \| \| 1.38E-17 \| \| 2.82E-17 \| \| 2.91E-17 \| \| 2.67E-16 \| \| 2.70E-16 \| \| 3.27E-16 \| \| 3.89E-16 \| \| 3.91E-15 \| \| 1.07E-14 \| \| 1.43E-14 \| \| 1.94E-14 \| \| 2.73E-14 \| \| 2.79E-14 \| \| 7.63E-14 \| \| 9.29E-14 \| \| 9.33E-14 \| \| 9.47E-14 \| \| 1.52E-13 \| \| 2.27E-13 \| \| 3.65E-13 \| \| 3.85E-13 \| \| 7.42E-13 \| \| 8.02E-13 \| \| 8.11E-13 \| \| 8.16E-13 \| \| 1.29E-12 \| \| 2.09E-12 \| \| 2.68E-12 \| \| 4.09E-12 \| \| 4.51E-12 \| \| 1.19E-11 \| \| 1.80E-11 \| \| 2.04E-11 \| \| 2.08E-11 \| \| 2.33E-11 \| \| 2.72E-11 \| \| 3.08E-11 \| \| 3.34E-11 \| \| 4.59E-11 \| \| 6.54E-11 \| \| 8.23E-11 \| \| 9.66E-11 \| \| 1.07E-10 \| \| 1.89E-10 \| \| 2.71E-10 \| \| 3.16E-10 \| \| 3.25E-10 \| \| 4.33E-10 \| \| 4.74E-10 \| \| 5.05E-10 \| \| 7.85E-10 \| \| 8.14E-10 \| \| 8.37E-10 \| \| 8.44E-10 \| \| 8.53E-10 \| \| 9.27E-10 \| \| 1.16E-09 \| \| 1.24E-09 \| \| 1.37E-09 \| \| 1.73E-09 \| \| 1.75E-09 \| \| 1.78E-09 \| \| 2.19E-09 \| \| 2.61E-09 \| \| 2.78E-09 \| \| 2.93E-09 \| \| 3.42E-09 \| \| 4.07E-09 \| \| 4.33E-09 \| \| 4.83E-09 \| \| 4.93E-09 \| \| 5.80E-09 \| \| 6.56E-09 \| \| 7.78E-09 \| \| 7.96E-09 \| \| 1.06E-08 \| \| 1.14E-08 \| \| 1.14E-08 \| \| 1.22E-08 \| \| 1.52E-08 \| \| 1.65E-08 \| \| 1.65E-08 \| \| 1.73E-08 \| \| 1.88E-08 \| \| 2.16E-08 \| \| 2.17E-08 \| \| 2.19E-08 \| \| 2.21E-08 \| \| 2.26E-08 \| \| 2.28E-08 \| \| 2.52E-08 \| \| 2.66E-08 \| \| 2.68E-08 \| \| 2.90E-08 \| \| 3.18E-08 \| \| 3.32E-08 \| \| 3.70E-08 \| \| 4.38E-08 \| \| 5.32E-08 \| \| 5.37E-08 \| \| 5.70E-08 \| \| 5.85E-08 \| \| 5.99E-08 \| \| 6.03E-08 \| \| 6.08E-08 \| \| 6.09E-08 \| \| 6.13E-08 \| \| 6.81E-08 \| \| 6.91E-08 \| \| 7.22E-08 \| \| 7.44E-08 \| \| 7.63E-08 \| \| 7.97E-08 \| \| 8.10E-08 \| \| 9.84E-08 \| \| 9.85E-08 \| \| 1.04E-07 \| \| 1.14E-07 \| \| 1.35E-07 \| \| 1.39E-07 \| \| 1.39E-07 \| \| 1.47E-07 \| \| 1.52E-07 \| \| 1.54E-07 \| \| 1.61E-07 \| \| 1.75E-07 \| \| 1.77E-07 \| \| 1.86E-07 \| \| 1.88E-07 \| \| 1.95E-07 \| \| 2.17E-07 \| \| 2.39E-07 \| \| 2.66E-07 \| \| 2.83E-07 \| \| 2.83E-07 \| \| 2.84E-07 \| \| 2.88E-07 \| \| 2.94E-07 \| \| 3.07E-07 \| \| 3.35E-07 \| \| 3.57E-07 \| \| 3.65E-07 \| \| 3.82E-07 \| \| 4.08E-07 \| \| 4.22E-07 \| \| 4.49E-07 \| \| 4.70E-07 \| \| 5.14E-07 \| \| 5.51E-07 \| \| 5.59E-07 \| \| 5.61E-07 \| \| 5.62E-07 \| \| 5.66E-07 \| \| 5.81E-07 \| \| 6.07E-07 \| \| 6.11E-07 \| \| 6.45E-07 \| \| 6.75E-07 \| \| 6.89E-07 \| \| 6.91E-07 \| \| 7.12E-07 \| \| 7.95E-07 \| \| 9.13E-07 \| \| 9.55E-07 \| \| 9.59E-07 \| \| 9.85E-07 \| \| 1.03E-06 \| \| 1.04E-06 \| \| 1.10E-06 \| \| 1.19E-06 \| \| 1.25E-06 \| \| 1.28E-06 \| \| 1.30E-06 \| \| 1.30E-06 \| \| 1.36E-06 \| \| 1.47E-06 \| \| 1.54E-06 \| \| 1.64E-06 \| \| 1.82E-06 \| \| 2.04E-06 \| \| 2.05E-06 \| \| 2.47E-06 \| \| 2.48E-06 \| \| 2.50E-06 \| \| 2.56E-06 \| \| 2.58E-06 \| \| 2.85E-06 \| \| 3.04E-06 \| \| 3.05E-06 \| \| 3.21E-06 \| \| 3.41E-06 \| \| 3.45E-06 \| \| 3.52E-06 \| \| 3.53E-06 \| \| 3.56E-06 \| \| 3.65E-06 \| \| 3.73E-06 \| \| 4.16E-06 \| \| 4.33E-06 \| \| 4.34E-06 \| \| 4.62E-06 \| \| 4.62E-06 \| \| 4.71E-06 \| \| 4.94E-06 \| \| 5.01E-06 \| \| 5.12E-06 \| \| 5.25E-06 \| \| 5.30E-06 \| \| 5.41E-06 \| \| 5.43E-06 \| \| 5.65E-06 \| \| 5.77E-06 \| \| 5.78E-06 \| \| 6.21E-06 \| \| 6.35E-06 \| \| 6.57E-06 \| \| 6.69E-06 \| \| 6.89E-06 \| \| 6.90E-06 \| \| 7.02E-06 \| \| 7.06E-06 \| \| 7.30E-06 \| \| 7.49E-06 \| \| 7.56E-06 \| \| 8.14E-06 \| \| 8.28E-06 \| \| 8.33E-06 \| \| 8.35E-06 \| \| 8.46E-06 \| \| 8.67E-06 \| \| 8.73E-06 \| \| 8.83E-06 \| \| 9.06E-06 \| \| 9.15E-06 \| \| 9.41E-06 \| \| 9.60E-06 \| \| 9.78E-06 \| \| 9.99E-06 \| \| 1.01E-05 \| \| 1.03E-05 \| \| 1.03E-05 \| \| 1.07E-05 \| \| 1.09E-05 \| \| 1.10E-05 \| \| 1.11E-05 \| \| 1.15E-05 \| \| 1.16E-05 \| \| 1.16E-05 \| \| 1.20E-05 \| \| 1.24E-05 \| \| 1.24E-05 \| \| 1.27E-05 \| \| 1.29E-05 \| \| 1.31E-05 \| \| 1.34E-05 \| \| 1.37E-05 \| \| 1.43E-05 \| \| 1.54E-05 \| \| 1.55E-05 \| \| 1.65E-05 \| \| 1.65E-05 \| \| 1.66E-05 \| \| 1.68E-05 \| \| 1.75E-05 \| \| 1.76E-05 \| \| 1.78E-05 \| \| 1.81E-05 \| \| 1.82E-05 \| \| 1.87E-05 \| \| 1.87E-05 \| \| 1.89E-05 \| \| 1.91E-05 \| \| 1.97E-05 \| \| 1.97E-05 \| \| 2.03E-05 \| \| 2.10E-05 \| \| 2.13E-05 \| \| 2.19E-05 \| \| 2.24E-05 \| \| 2.25E-05 \| \| 2.27E-05 \| \| 2.27E-05 \| \| 2.32E-05 \| \| 2.36E-05 \| \| 2.37E-05 \| \| 2.39E-05 \| \| 2.47E-05 \| \| 2.49E-05 \| \| 2.60E-05 \| \| 2.65E-05 \| \| 2.71E-05 \| \| 2.86E-05 \| \| 2.87E-05 \| \| 2.87E-05 \| \| 3.07E-05 \| \| 3.08E-05 \| \| 3.15E-05 \| \| 3.28E-05 \| \| 3.29E-05 \| \| 3.31E-05 \| \| 3.32E-05 \| \| 3.40E-05 \| \| 3.45E-05 \| \| 3.55E-05 \| \| 3.57E-05 \| \| 3.61E-05 \| \| 3.74E-05 \| \| 3.83E-05 \| \| 3.95E-05 \| \| 3.98E-05 \| \| 3.99E-05 \| \| 4.14E-05 \| \| 4.17E-05 \| \| 4.19E-05 \| \| 4.23E-05 \| \| 4.26E-05 \| \| 4.39E-05 \| \| 4.45E-05 \| \| 4.51E-05 \| \| 4.73E-05 \| \| 4.88E-05 \| \| 5.10E-05 \| \| 5.16E-05 \| \| 5.21E-05 \| \| 5.25E-05 \| \| 5.25E-05 \| \| 5.29E-05 \| \| 5.33E-05 \| \| 5.52E-05 \| \| 5.53E-05 \| \| 5.54E-05 \| \| 5.61E-05 \| \| 5.64E-05 \| \| 5.81E-05 \| \| 5.86E-05 \| \| 5.95E-05 \| \| 5.96E-05 \| \| 5.99E-05 \| \| 6.00E-05 \| \| 6.02E-05 \| \| 6.05E-05 \| \| 6.10E-05 \| \| 6.15E-05 \| \| 6.15E-05 \| \| 6.38E-05 \| \| 6.48E-05 \| \| 6.53E-05 \| \| 6.64E-05 \| \| 6.70E-05 \| \| 6.74E-05 \| \| 6.88E-05 \| \| 6.93E-05 \| \| 6.95E-05 \| \| 6.97E-05 \| \| 7.06E-05 \| \| 7.25E-05 \| \| 7.39E-05 \| \| 7.76E-05 \| \| 7.95E-05 \| \| 7.98E-05 \| \| 8.00E-05 \| \| 8.06E-05 \| \| 8.10E-05 \| \| 8.52E-05 \| \| 8.55E-05 \| \| 8.58E-05 \| \| 8.63E-05 \| \| 8.64E-05 \| \| 8.64E-05 \| \| 8.67E-05 \| \| 8.90E-05 \| \| 9.08E-05 \| \| 9.16E-05 \| \| 9.17E-05 \| \| 9.42E-05 \| \| 9.56E-05 \| \| 9.58E-05 \| \| 9.81E-05 \| \| 9.85E-05 \| \| 0.000100216 \| \| 0.000100804 \| \| 0.000102768 \| \| 0.000103504 \| \| 0.000105537 \| \| 0.000105757 \| \| 0.000106322 \| \| 0.000109667 \| \| 0.000110685 \| \| 0.000111249 \| \| 0.000111624 \| \| 0.000112363 \| \| 0.000114068 \| \| 0.000114336 \| \| 0.000116213 \| \| 0.000116275 \| \| 0.00011759 \| \| 0.00011901 \| \| 0.000119705 \| \| 0.000122212 \| \| 0.000128752 \| \| 0.000129535 \| \| 0.000131305 \| \| 0.000133282 \| \| 0.000133358 \| \| 0.000134241 \| \| 0.000134376 \| \| 0.000136389 \| \| 0.00013825 \| \| 0.000138884 \| \| 0.000140729 \| \| 0.000143468 \| \| 0.000144486 \| \| 0.000148385 \| \| 0.000148718 \| \| 0.000149028 \| \| 0.000155407 \| \| 0.000158433 \| \| 0.000160866 \| \| 0.000166188 \| \| 0.000169122 \| \| 0.000173881 \| \| 0.00017518 \| \| 0.000176144 \| \| 0.000177848 \| \| 0.000182719 \| \| 0.000182744 \| \| 0.000182915 \| \| 0.000184534 \| \| 0.000186454 \| \| 0.000191876 \| \| 0.000195112 \| \| 0.0001955 \| \| 0.000198841 \| \| 0.000200529 \| \| 0.000202273 \| \| 0.000203708 \| \| 0.000205759 \| \| 0.00020759 \| \| 0.000210577 \| \| 0.000210947 \| \| 0.000214389 \| \| 0.000215236 \| \| 0.000215282 \| \| 0.000219235 \| \| 0.000221746 \| \| 0.000224365 \| \| 0.0002249 \| \| 0.000230226 \| \| 0.000230828 \| \| 0.0002313 \| \| 0.000233588 \| \| 0.000237079 \| \| 0.000238168 \| \| 0.000239366 \| \| 0.000247184 \| \| 0.000247913 \| \| 0.000257911 \| \| 0.00026022 \| \| 0.000261498 \| \| 0.00026858 \| \| 0.000273054 \| \| 0.000273915 \| \| 0.000279281 \| \| 0.000279923 \| \| 0.000282605 \| \| 0.000288421 \| \| 0.000292331 \| \| 0.000297853 \| \| 0.000299002 \| \| 0.000299657 \| \| 0.000302245 \| \| 0.000302545 \| \| 0.000304412 \| \| 0.000308826 \| \| 0.000311912 \| \| 0.000315201 \| \| 0.000316518 \| \| 0.000321794 \| \| 0.000328355 \| \| 0.000328681 \| \| 0.000329154 \| \| 0.000329857 \| \| 0.00033205 \| \| 0.000334669 \| \| 0.000334766 \| \| 0.000336012 \| \| 0.000349026 \| \| 0.000349488 \| \| 0.000350385 \| \| 0.000352779 \| \| 0.000353273 \| \| 0.000355173 \| \| 0.000360297 \| \| 0.000361818 \| \| 0.000367601 \| \| 0.000371047 \| \| 0.000374962 \| \| 0.000380477 \| \| 0.00038269 \| \| 0.000383061 \| \| 0.000389222 \| \| 0.000390352 \| \| 0.000391696 \| \| 0.000393787 \| \| 0.000395114 \| \| 0.000396521 \| \| 0.000396708 \| \| 0.000398022 \| \| 0.000398928 \| \| 0.000399358 \| \| 0.000400277 \| \| 0.000419692 \| \| 0.000421879 \| \| 0.000422199 \| \| 0.000422602 \| \| 0.00042498 \| \| 0.00042907 \| \| 0.000434303 \| \| 0.000449347 \| \| 0.000466032 \| \| 0.000471946 \| \| 0.000478758 \| \| 0.000478922 \| \| 0.000479234 \| \| 0.000488126 \| \| 0.000495317 \| \| 0.000495571 \| \| 0.000496723 \| \| 0.000497916 \| \| 0.000502838 \| \| 0.000503058 \| \| 0.000504699 \| \| 0.00051072 \| \| 0.000513713 \| \| 0.00052119 \| \| 0.000523023 \| \| 0.000527534 \| \| 0.000531204 \| \| 0.000538338 \| \| 0.00053863 \| \| 0.000542294 \| \| 0.000547769 \| \| 0.000549738 \| \| 0.000558521 \| \| 0.000558884 \| \| 0.000559274 \| \| 0.000559659 \| \| 0.000563314 \| \| 0.000563426 \| \| 0.000565634 \| \| 0.000566336 \| \| 0.000576879 \| \| 0.000591117 \| \| 0.000591569 \| \| 0.000601771 \| \| 0.000608926 \| \| 0.000611491 \| \| 0.000616946 \| \| 0.00061839 \| \| 0.000630392 \| \| 0.000632785 \| \| 0.000633855 \| \| 0.000636506 \| \| 0.000638811 \| \| 0.000655245 \| \| 0.00065689 \| \| 0.000660642 \| \| 0.000665358 \| \| 0.000675313 \| \| 0.000687606 \| \| 0.000690421 \| \| 0.00069203 \| \| 0.000704601 \| \| 0.000707584 \| \| 0.000711609 \| \| 0.0007202 \| \| 0.000723519 \| \| 0.000728478 \| \| 0.000737978 \| \| 0.000748415 \| \| 0.000750653 \| \| 0.000754714 \| \| 0.000760063 \| \| 0.000760627 \| \| 0.000768975 \| \| 0.000776011 \| \| 0.000780349 \| \| 0.000780664 \| \| 0.000782905 \| \| 0.000786606 \| \| 0.000794624 \| \| 0.000797145 \| \| 0.000797773 \| \| 0.000803506 \| \| 0.000812673 \| \| 0.000823635 \| \| 0.000831885 \| \| 0.000832365 \| \| 0.000835346 \| \| 0.000844031 \| \| 0.000847187 \| \| 0.000851394 \| \| 0.000866361 \| \| 0.000867132 \| \| 0.000869804 \| \| 0.000876528 \| \| 0.000884539 \| \| 0.000896593 \| \| 0.000903269 \| \| 0.000910398 \| \| 0.000916034 \| \| 0.000923436 \| \| 0.000925337 \| \| 0.00093304 \| \| 0.00093912 \| \| 0.000942997 \| \| 0.000948292 \| \| 0.000952731 \| \| 0.00097881 \| \| 0.000993627 \| \| 0.001002685 \| \| 0.001014137 \| \| 0.001022592 \| \| 0.001022803 \| \| 0.001064509 \| \| 0.001066727 \| \| 0.001076177 \| \| 0.001082131 \| \| 0.00108587 \| \| 0.001089315 \| \| 0.00109392 \| \| 0.001093965 \| \| 0.001098762 \| \| 0.001103688 \| \| 0.001103692 \| \| 0.00110761 \| \| 0.00110811 \| \| 0.001117238 \| \| 0.001121543 \| \| 0.001123825 \| \| 0.001132012 \| \| 0.001134851 \| \| 0.001149371 \| \| 0.001149584 \| \| 0.001177936 \| \| 0.001179568 \| \| 0.001182121 \| \| 0.001196582 \| \| 0.001220388 \| \| 0.001222643 \| \| 0.001244822 \| \| 0.001263313 \| \| 0.001265066 \| \| 0.001279737 \| \| 0.00128856 \| \| 0.00128967 \| \| 0.001291591 \| \| 0.001298931 \| \| 0.001301798 \| \| 0.001302316 \| \| 0.001309644 \| \| 0.001312041 \| \| 0.001319426 \| \| 0.001323939 \| \| 0.00132534 \| \| 0.001348498 \| \| 0.001355285 \| \| 0.001356033 \| \| 0.001366075 \| \| 0.001366967 \| \| 0.001400611 \| \| 0.001404873 \| \| 0.001409369 \| \| 0.001418264 \| \| 0.001421205 \| \| 0.001426304 \| \| 0.001435279 \| \| 0.001441469 \| \| 0.001453458 \| \| 0.001459158 \| \| 0.001465634 \| \| 0.001468488 \| \| 0.001476952 \| \| 0.001483048 \| \| 0.001497197 \| \| 0.001514375 \| \| 0.001523145 \| \| 0.001527425 \| \| 0.001529009 \| \| 0.001534016 \| \| 0.001541029 \| \| 0.001541361 \| \| 0.001558508 \| \| 0.001567637 \| \| 0.001580995 \| \| 0.00158692 \| \| 0.001588125 \| \| 0.001588654 \| \| 0.001592216 \| \| 0.001613844 \| \| 0.001622326 \| \| 0.00162955 \| \| 0.0016302 \| \| 0.00163315 \| \| 0.001639477 \| \| 0.001653552 \| \| 0.001679427 \| \| 0.001703326 \| \| 0.001709801 \| \| 0.001712933 \| \| 0.001718182 \| \| 0.001725783 \| \| 0.00172993 \| \| 0.001742153 \| \| 0.001764159 \| \| 0.001767721 \| \| 0.00177201 \| \| 0.001800675 \| \| 0.001801894 \| \| 0.001823019 \| \| 0.001823927 \| \| 0.001832077 \| \| 0.001836846 \| \| 0.00183855 \| \| 0.00184692 \| \| 0.001857823 \| \| 0.001866467 \| \| 0.001869592 \| \| 0.001903071 \| \| 0.001908014 \| \| 0.001911193 \| \| 0.001912732 \| \| 0.001916649 \| \| 0.001918424 \| \| 0.001942037 \| \| 0.001942368 \| \| 0.001947208 \| \| 0.0019478 \| \| 0.001953651 \| \| 0.001970445 \| \| 0.00199487 \| \| 0.001996364 \| \| 0.00202482 \| \| 0.002027121 \| \| 0.002029642 \| \| 0.002037857 \| \| 0.002041727 \| \| 0.002048782 \| \| 0.002054317 \| \| 0.002055084 \| \| 0.002061821 \| \| 0.002087818 \| \| 0.002094034 \| \| 0.002106908 \| \| 0.002108887 \| \| 0.00211137 \| \| 0.002119433 \| \| 0.002120287 \| \| 0.002131709 \| \| 0.002142388 \| \| 0.002142924 \| \| 0.002146461 \| \| 0.002153294 \| \| 0.002157837 \| \| 0.002167048 \| \| 0.002179937 \| \| 0.002191761 \| \| 0.002213162 \| \| 0.002238337 \| \| 0.002250468 \| \| 0.002273675 \| \| 0.002279522 \| \| 0.002303444 \| \| 0.002309184 \| \| 0.002314441 \| \| 0.002329582 \| \| 0.002333206 \| \| 0.002354243 \| \| 0.002358997 \| \| 0.002370605 \| \| 0.002370647 \| \| 0.00237219 \| \| 0.002414213 \| \| 0.002436721 \| \| 0.002440454 \| | \| 2.47E-43 \| \| --- \| \| 1.18E-36 \| \| 1.17E-30 \| \| 1.91E-29 \| \| 4.33E-26 \| \| 2.11E-24 \| \| 2.11E-24 \| \| 3.33E-22 \| \| 3.88E-20 \| \| 3.33E-19 \| \| 1.10E-18 \| \| 6.94E-18 \| \| 9.27E-18 \| \| 1.22E-17 \| \| 2.51E-17 \| \| 1.88E-16 \| \| 3.80E-16 \| \| 6.28E-16 \| \| 4.93E-15 \| \| 1.12E-14 \| \| 2.15E-14 \| \| 2.15E-14 \| \| 1.83E-13 \| \| 1.83E-13 \| \| 2.13E-13 \| \| 2.44E-13 \| \| 2.36E-12 \| \| 6.21E-12 \| \| 8.05E-12 \| \| 1.05E-11 \| \| 1.42E-11 \| \| 1.42E-11 \| \| 3.76E-11 \| \| 4.28E-11 \| \| 4.28E-11 \| \| 4.28E-11 \| \| 6.68E-11 \| \| 9.73E-11 \| \| 1.52E-10 \| \| 1.57E-10 \| \| 2.94E-10 \| \| 3.02E-10 \| \| 3.02E-10 \| \| 3.02E-10 \| \| 4.65E-10 \| \| 7.41E-10 \| \| 9.27E-10 \| \| 1.39E-09 \| \| 1.50E-09 \| \| 3.89E-09 \| \| 5.73E-09 \| \| 6.38E-09 \| \| 6.39E-09 \| \| 7.03E-09 \| \| 8.05E-09 \| \| 8.96E-09 \| \| 9.52E-09 \| \| 1.29E-08 \| \| 1.80E-08 \| \| 2.23E-08 \| \| 2.58E-08 \| \| 2.80E-08 \| \| 4.89E-08 \| \| 6.88E-08 \| \| 7.90E-08 \| \| 8.00E-08 \| \| 1.05E-07 \| \| 1.14E-07 \| \| 1.19E-07 \| \| 1.82E-07 \| \| 1.86E-07 \| \| 1.87E-07 \| \| 1.87E-07 \| \| 1.87E-07 \| \| 2.01E-07 \| \| 2.48E-07 \| \| 2.62E-07 \| \| 2.85E-07 \| \| 3.56E-07 \| \| 3.56E-07 \| \| 3.58E-07 \| \| 4.34E-07 \| \| 5.13E-07 \| \| 5.39E-07 \| \| 5.61E-07 \| \| 6.47E-07 \| \| 7.61E-07 \| \| 8.00E-07 \| \| 8.84E-07 \| \| 8.92E-07 \| \| 1.04E-06 \| \| 1.16E-06 \| \| 1.36E-06 \| \| 1.38E-06 \| \| 1.82E-06 \| \| 1.92E-06 \| \| 1.92E-06 \| \| 2.03E-06 \| \| 2.50E-06 \| \| 2.66E-06 \| \| 2.66E-06 \| \| 2.77E-06 \| \| 2.97E-06 \| \| 3.36E-06 \| \| 3.36E-06 \| \| 3.36E-06 \| \| 3.37E-06 \| \| 3.41E-06 \| \| 3.41E-06 \| \| 3.73E-06 \| \| 3.89E-06 \| \| 3.89E-06 \| \| 4.17E-06 \| \| 4.54E-06 \| \| 4.70E-06 \| \| 5.19E-06 \| \| 6.10E-06 \| \| 7.33E-06 \| \| 7.34E-06 \| \| 7.74E-06 \| \| 7.87E-06 \| \| 7.92E-06 \| \| 7.92E-06 \| \| 7.92E-06 \| \| 7.92E-06 \| \| 7.92E-06 \| \| 8.72E-06 \| \| 8.78E-06 \| \| 9.11E-06 \| \| 9.32E-06 \| \| 9.47E-06 \| \| 9.83E-06 \| \| 9.90E-06 \| \| 1.19E-05 \| \| 1.19E-05 \| \| 1.25E-05 \| \| 1.35E-05 \| \| 1.59E-05 \| \| 1.62E-05 \| \| 1.62E-05 \| \| 1.70E-05 \| \| 1.74E-05 \| \| 1.75E-05 \| \| 1.82E-05 \| \| 1.97E-05 \| \| 1.97E-05 \| \| 2.06E-05 \| \| 2.06E-05 \| \| 2.13E-05 \| \| 2.35E-05 \| \| 2.58E-05 \| \| 2.84E-05 \| \| 2.99E-05 \| \| 2.99E-05 \| \| 2.99E-05 \| \| 3.00E-05 \| \| 3.05E-05 \| \| 3.16E-05 \| \| 3.43E-05 \| \| 3.63E-05 \| \| 3.68E-05 \| \| 3.84E-05 \| \| 4.07E-05 \| \| 4.19E-05 \| \| 4.43E-05 \| \| 4.61E-05 \| \| 5.01E-05 \| \| 5.34E-05 \| \| 5.35E-05 \| \| 5.35E-05 \| \| 5.35E-05 \| \| 5.35E-05 \| \| 5.46E-05 \| \| 5.67E-05 \| \| 5.68E-05 \| \| 5.96E-05 \| \| 6.20E-05 \| \| 6.28E-05 \| \| 6.28E-05 \| \| 6.44E-05 \| \| 7.15E-05 \| \| 8.16E-05 \| \| 8.48E-05 \| \| 8.48E-05 \| \| 8.66E-05 \| \| 8.97E-05 \| \| 9.06E-05 \| \| 9.54E-05 \| \| 0.000102191 \| \| 0.000107202 \| \| 0.000108946 \| \| 0.000109413 \| \| 0.000109413 \| \| 0.000113662 \| \| 0.000123006 \| \| 0.000127642 \| \| 0.00013579 \| \| 0.000149774 \| \| 0.000166663 \| \| 0.000167044 \| \| 0.000199569 \| \| 0.000199569 \| \| 0.000200704 \| \| 0.000204433 \| \| 0.000204553 \| \| 0.000225072 \| \| 0.000238746 \| \| 0.000238746 \| \| 0.000249822 \| \| 0.000264147 \| \| 0.000265669 \| \| 0.000269939 \| \| 0.000269939 \| \| 0.000270361 \| \| 0.000276342 \| \| 0.000281078 \| \| 0.000311845 \| \| 0.000322514 \| \| 0.000322514 \| \| 0.000340007 \| \| 0.000340007 \| \| 0.000345143 \| \| 0.00036008 \| \| 0.000363854 \| \| 0.000370507 \| \| 0.000378279 \| \| 0.000379926 \| \| 0.000386129 \| \| 0.000386129 \| \| 0.000399562 \| \| 0.000405255 \| \| 0.000405255 \| \| 0.000433345 \| \| 0.000441214 \| \| 0.000454771 \| \| 0.000460996 \| \| 0.000471461 \| \| 0.000471461 \| \| 0.000478029 \| \| 0.000478356 \| \| 0.00049311 \| \| 0.00050368 \| \| 0.000505893 \| \| 0.000543077 \| \| 0.000550093 \| \| 0.000550214 \| \| 0.000550214 \| \| 0.000554931 \| \| 0.000566481 \| \| 0.000568338 \| \| 0.000572379 \| \| 0.000585027 \| \| 0.000588509 \| \| 0.00060305 \| \| 0.000612584 \| \| 0.000621885 \| \| 0.00063239 \| \| 0.000638401 \| \| 0.000645814 \| \| 0.000645814 \| \| 0.000668899 \| \| 0.000678889 \| \| 0.000678889 \| \| 0.000686639 \| \| 0.000708033 \| \| 0.000709422 \| \| 0.000709422 \| \| 0.000730648 \| \| 0.000748382 \| \| 0.000749521 \| \| 0.000762375 \| \| 0.000772009 \| \| 0.000781993 \| \| 0.000795067 \| \| 0.000810627 \| \| 0.000841848 \| \| 0.000901988 \| \| 0.000906352 \| \| 0.000957905 \| \| 0.000957905 \| \| 0.000958945 \| \| 0.00096968 \| \| 0.001005859 \| \| 0.001008213 \| \| 0.001014987 \| \| 0.001029423 \| \| 0.001029423 \| \| 0.001052219 \| \| 0.001052219 \| \| 0.001059722 \| \| 0.001067436 \| \| 0.001094014 \| \| 0.001094014 \| \| 0.00112115 \| \| 0.0011601 \| \| 0.001170183 \| \| 0.00119738 \| \| 0.001222137 \| \| 0.001225157 \| \| 0.001228047 \| \| 0.001228047 \| \| 0.001247489 \| \| 0.001268996 \| \| 0.001269132 \| \| 0.001272879 \| \| 0.001314182 \| \| 0.001319127 \| \| 0.001375292 \| \| 0.001396928 \| \| 0.001421223 \| \| 0.001492611 \| \| 0.001492611 \| \| 0.001492611 \| \| 0.001592461 \| \| 0.001593037 \| \| 0.001620167 \| \| 0.001682198 \| \| 0.001682198 \| \| 0.001690041 \| \| 0.001690488 \| \| 0.00172337 \| \| 0.001745257 \| \| 0.001786383 \| \| 0.001792384 \| \| 0.001805277 \| \| 0.001868962 \| \| 0.001905603 \| \| 0.001958365 \| \| 0.001968424 \| \| 0.001968424 \| \| 0.002035222 \| \| 0.002045019 \| \| 0.002046567 \| \| 0.002061447 \| \| 0.002068964 \| \| 0.002126893 \| \| 0.002148935 \| \| 0.002170017 \| \| 0.002272463 \| \| 0.002337146 \| \| 0.002435677 \| \| 0.002455269 \| \| 0.002473819 \| \| 0.002475252 \| \| 0.002475252 \| \| 0.00248864 \| \| 0.002499237 \| \| 0.002577185 \| \| 0.002577185 \| \| 0.002577507 \| \| 0.00260119 \| \| 0.002605159 \| \| 0.002677709 \| \| 0.002691514 \| \| 0.002725298 \| \| 0.002725298 \| \| 0.002728966 \| \| 0.002728966 \| \| 0.002730109 \| \| 0.002735524 \| \| 0.002751594 \| \| 0.002758063 \| \| 0.002758063 \| \| 0.002851023 \| \| 0.002889024 \| \| 0.002901798 \| \| 0.002943203 \| \| 0.002961099 \| \| 0.002970943 \| \| 0.003027658 \| \| 0.003039405 \| \| 0.003039405 \| \| 0.003041372 \| \| 0.003071304 \| \| 0.003144339 \| \| 0.00319767 \| \| 0.003350213 \| \| 0.003421228 \| \| 0.003423992 \| \| 0.003426635 \| \| 0.003440695 \| \| 0.003450441 \| \| 0.003620852 \| \| 0.003622955 \| \| 0.003622955 \| \| 0.003622955 \| \| 0.003622955 \| \| 0.003622955 \| \| 0.00362525 \| \| 0.003714354 \| \| 0.0037774 \| \| 0.003794672 \| \| 0.003794672 \| \| 0.003892087 \| \| 0.003934354 \| \| 0.003934354 \| \| 0.004021499 \| \| 0.004027472 \| \| 0.004086763 \| \| 0.004100459 \| \| 0.00416992 \| \| 0.004189329 \| \| 0.004259343 \| \| 0.004259343 \| \| 0.004271526 \| \| 0.004395047 \| \| 0.004424943 \| \| 0.004436613 \| \| 0.004440673 \| \| 0.00445917 \| \| 0.004515421 \| \| 0.004515421 \| \| 0.004569838 \| \| 0.004569838 \| \| 0.004610379 \| \| 0.004654852 \| \| 0.004670802 \| \| 0.004757223 \| \| 0.004999819 \| \| 0.00501823 \| \| 0.005074749 \| \| 0.005129712 \| \| 0.005129712 \| \| 0.005144562 \| \| 0.005144562 \| \| 0.005209341 \| \| 0.005268077 \| \| 0.005279853 \| \| 0.005337517 \| \| 0.005428779 \| \| 0.005454596 \| \| 0.005587169 \| \| 0.005587169 \| \| 0.005587169 \| \| 0.005812952 \| \| 0.005912547 \| \| 0.005989582 \| \| 0.006173611 \| \| 0.006268296 \| \| 0.006430037 \| \| 0.006463383 \| \| 0.00648425 \| \| 0.0065322 \| \| 0.006673099 \| \| 0.006673099 \| \| 0.006673099 \| \| 0.006717111 \| \| 0.006771851 \| \| 0.00695326 \| \| 0.007053167 \| \| 0.007053167 \| \| 0.007157854 \| \| 0.007202659 \| \| 0.007249306 \| \| 0.00728468 \| \| 0.007341895 \| \| 0.007391033 \| \| 0.00747782 \| \| 0.00747782 \| \| 0.007581928 \| \| 0.007581928 \| \| 0.007581928 \| \| 0.007704472 \| \| 0.007775922 \| \| 0.007850843 \| \| 0.007852688 \| \| 0.008021413 \| \| 0.008024494 \| \| 0.008024494 \| \| 0.008086604 \| \| 0.008190054 \| \| 0.008210251 \| \| 0.008234086 \| \| 0.008485084 \| \| 0.008492211 \| \| 0.008816098 \| \| 0.008876398 \| \| 0.008901336 \| \| 0.009123309 \| \| 0.009255946 \| \| 0.009265851 \| \| 0.009427767 \| \| 0.009429874 \| \| 0.009500566 \| \| 0.009676078 \| \| 0.009787088 \| \| 0.009951481 \| \| 0.009969384 \| \| 0.009970811 \| \| 0.01002588 \| \| 0.01002588 \| \| 0.010067236 \| \| 0.010192509 \| \| 0.010273508 \| \| 0.010360873 \| \| 0.010383207 \| \| 0.010535015 \| \| 0.010711339 \| \| 0.010711339 \| \| 0.010711339 \| \| 0.010712788 \| \| 0.010762508 \| \| 0.010807488 \| \| 0.010807488 \| \| 0.010826227 \| \| 0.011216013 \| \| 0.011216013 \| \| 0.011222671 \| \| 0.01127079 \| \| 0.01127079 \| \| 0.011309227 \| \| 0.011449972 \| \| 0.011475903 \| \| 0.01163664 \| \| 0.01172292 \| \| 0.011823658 \| \| 0.011974351 \| \| 0.012009206 \| \| 0.012009206 \| \| 0.012178916 \| \| 0.012190818 \| \| 0.012209362 \| \| 0.012251068 \| \| 0.012268894 \| \| 0.012271544 \| \| 0.012271544 \| \| 0.012283475 \| \| 0.012283475 \| \| 0.012283475 \| \| 0.012288516 \| \| 0.012860281 \| \| 0.012876693 \| \| 0.012876693 \| \| 0.012876693 \| \| 0.012924957 \| \| 0.013025 \| \| 0.013159298 \| \| 0.013589838 \| \| 0.014068291 \| \| 0.014220444 \| \| 0.014360246 \| \| 0.014360246 \| \| 0.014360246 \| \| 0.014599814 \| \| 0.014768183 \| \| 0.014768183 \| \| 0.014775465 \| \| 0.014783918 \| \| 0.014882277 \| \| 0.014882277 \| \| 0.014903728 \| \| 0.015054199 \| \| 0.015115053 \| \| 0.015307368 \| \| 0.015333539 \| \| 0.015437957 \| \| 0.015517457 \| \| 0.015678081 \| \| 0.015678081 \| \| 0.015756533 \| \| 0.015887264 \| \| 0.015915981 \| \| 0.016088725 \| \| 0.016088725 \| \| 0.016088725 \| \| 0.016088725 \| \| 0.01613997 \| \| 0.01613997 \| \| 0.01616642 \| \| 0.01616642 \| \| 0.016438517 \| \| 0.0167983 \| \| 0.0167983 \| \| 0.017058212 \| \| 0.017231013 \| \| 0.017273566 \| \| 0.017397454 \| \| 0.017407999 \| \| 0.017715225 \| \| 0.017751215 \| \| 0.017751215 \| \| 0.017794816 \| \| 0.017828621 \| \| 0.018255979 \| \| 0.018270515 \| \| 0.018343524 \| \| 0.018442994 \| \| 0.018687111 \| \| 0.018994984 \| \| 0.019040403 \| \| 0.019052479 \| \| 0.019365821 \| \| 0.019414995 \| \| 0.019492567 \| \| 0.019694747 \| \| 0.019752319 \| \| 0.019854381 \| \| 0.020079661 \| \| 0.020329641 \| \| 0.020356445 \| \| 0.020432521 \| \| 0.020524317 \| \| 0.020524317 \| \| 0.020715231 \| \| 0.020870193 \| \| 0.020926158 \| \| 0.020926158 \| \| 0.020951711 \| \| 0.021016199 \| \| 0.021195613 \| \| 0.021210069 \| \| 0.021210069 \| \| 0.021327649 \| \| 0.021535822 \| \| 0.021790847 \| \| 0.021950417 \| \| 0.021950417 \| \| 0.021993401 \| \| 0.022186144 \| \| 0.022233188 \| \| 0.022307622 \| \| 0.022647034 \| \| 0.022647034 \| \| 0.022680409 \| \| 0.022819173 \| \| 0.022990958 \| \| 0.023267077 \| \| 0.023403018 \| \| 0.023550215 \| \| 0.023658394 \| \| 0.023811769 \| \| 0.023823024 \| \| 0.023983395 \| \| 0.024101622 \| \| 0.024163006 \| \| 0.024260477 \| \| 0.024335768 \| \| 0.024962713 \| \| 0.025300941 \| \| 0.025491708 \| \| 0.02574262 \| \| 0.025881856 \| \| 0.025881856 \| \| 0.02689538 \| \| 0.026909635 \| \| 0.027105996 \| \| 0.027213833 \| \| 0.027265736 \| \| 0.027310094 \| \| 0.027342392 \| \| 0.027342392 \| \| 0.027420176 \| \| 0.027458971 \| \| 0.027458971 \| \| 0.027484854 \| \| 0.027484854 \| \| 0.02766907 \| \| 0.027733485 \| \| 0.027747744 \| \| 0.027907517 \| \| 0.02793518 \| \| 0.0282125 \| \| 0.0282125 \| \| 0.02886127 \| \| 0.02886127 \| \| 0.02888031 \| \| 0.029189772 \| \| 0.029725948 \| \| 0.029736347 \| \| 0.030230599 \| \| 0.030630794 \| \| 0.030630794 \| \| 0.030939974 \| \| 0.031087732 \| \| 0.031087732 \| \| 0.031087994 \| \| 0.03120764 \| \| 0.03120764 \| \| 0.03120764 \| \| 0.03133708 \| \| 0.031348329 \| \| 0.031478571 \| \| 0.031527212 \| \| 0.031527212 \| \| 0.032031247 \| \| 0.032116459 \| \| 0.032116459 \| \| 0.032281461 \| \| 0.032281461 \| \| 0.033028041 \| \| 0.033080582 \| \| 0.033138491 \| \| 0.033299539 \| \| 0.033320493 \| \| 0.033391927 \| \| 0.033553761 \| \| 0.033650125 \| \| 0.033881389 \| \| 0.033965609 \| \| 0.034067605 \| \| 0.034085252 \| \| 0.03423288 \| \| 0.034325272 \| \| 0.034603538 \| \| 0.034950917 \| \| 0.035103535 \| \| 0.035139125 \| \| 0.035139125 \| \| 0.035204486 \| \| 0.035273526 \| \| 0.035273526 \| \| 0.035615852 \| \| 0.035774227 \| \| 0.036028526 \| \| 0.036051588 \| \| 0.036051588 \| \| 0.036051588 \| \| 0.036082094 \| \| 0.036521352 \| \| 0.036662309 \| \| 0.036738195 \| \| 0.036738195 \| \| 0.036753791 \| \| 0.036845207 \| \| 0.037110265 \| \| 0.037639058 \| \| 0.038122168 \| \| 0.038214525 \| \| 0.038232005 \| \| 0.038296624 \| \| 0.038413416 \| \| 0.03845314 \| \| 0.038671991 \| \| 0.039107135 \| \| 0.039132766 \| \| 0.039174426 \| \| 0.039727126 \| \| 0.039727126 \| \| 0.040104219 \| \| 0.040104219 \| \| 0.04022905 \| \| 0.040262514 \| \| 0.040262514 \| \| 0.040391454 \| \| 0.04057535 \| \| 0.040709505 \| \| 0.04072307 \| \| 0.041396873 \| \| 0.041440825 \| \| 0.041440825 \| \| 0.041440825 \| \| 0.041453755 \| \| 0.041453755 \| \| 0.041859957 \| \| 0.041859957 \| \| 0.041866123 \| \| 0.041866123 \| \| 0.041936475 \| \| 0.042241242 \| \| 0.042684421 \| \| 0.042684421 \| \| 0.043225537 \| \| 0.043225537 \| \| 0.043225537 \| \| 0.043343755 \| \| 0.043369373 \| \| 0.043462502 \| \| 0.043482796 \| \| 0.043482796 \| \| 0.043568692 \| \| 0.044060822 \| \| 0.044134746 \| \| 0.044327863 \| \| 0.044327863 \| \| 0.044327863 \| \| 0.04440051 \| \| 0.04440051 \| \| 0.044582307 \| \| 0.044701953 \| \| 0.044701953 \| \| 0.044718391 \| \| 0.04480338 \| \| 0.04484057 \| \| 0.044974543 \| \| 0.0451844 \| \| 0.045371682 \| \| 0.045756489 \| \| 0.046218253 \| \| 0.04640984 \| \| 0.046829081 \| \| 0.046890142 \| \| 0.047322393 \| \| 0.047380504 \| \| 0.047428544 \| \| 0.047678788 \| \| 0.047692955 \| \| 0.048062594 \| \| 0.048099296 \| \| 0.048187152 \| \| 0.048187152 \| \| 0.048187152 \| \| 0.048979618 \| \| 0.04937471 \| \| 0.049388837 \| |
|  |  |  |  |  |

**Supplementary Table4. Primer sequences**

| Primer name | Sequence |
| --- | --- |
| Gapdh-F | AATGGATTTGGACGCATTGGT |
| Gapdh-R | TTTGCACTGGTACGTGTTGAT |
| Lgr5-F | ACATTCCCAAGGGAGCGTTC |
| Lgr5-R | ATGTGGTTGGCATCTAGGCG |
| Ostn-F | CGTCTTGATGATCTGGTGTCC |
| Ostn-R | TGGGAATACCAAACCGCTTTT |
| Gdf10-F | CAGGACATGGTCGCTATCCAC |
| Gdf10-R | ACAGGCTTTTGGTCGATCATTTC |
| Tgfβ3-F | GGACTTCGGCCACATCAAGAA |
| Tgfβ3-R | TAGGGGACGTGGGTCATCAC |
| Wif1-F | GGCGAGAACTTCACAAGCAGC |
| Wif1-R | CAGCAGGAGCAGGCAAGGTAG |
| Foxp1-R | CCTCGCTCAAGGCATGATTC |
| Foxp1-F | GTGCTGGTCAGGTCTAGGC |
| Thy1-F | TGCTCTCAGTCTTGCAGGTG |
| Thy1-R | TGGATGGAGTTATCCTTGGTGTT |
| Sox11-F | CGAGCCTGTACGACGAAGTG |
| Sox11-R | AAGCTCAGGTCGAACATGAGG |
| Sox9-F | AGAAAGACCACCCCGATTACA |
| Sox9-R | AGCGCCTTGAAGATAGCATTA |
| Wnt11-F | ATGCGTCTACACAACAGTGAAG |
| Wnt11-R | GTAGCGGGTCTTGAGGTCAG |
| Frzb-F | GCTGTGCAAGTCCCTTCCC |
| Frzb-R | TGCAAATGGGTGCGTACATTG |
| Col2a1-F | AGCAAGAGCAAGGAAAAGAAA |
| Col2a1-R | GTGGACAGTAGACGGAGGAAA |
| Ihh-F | CTCTTGCCTACAAGCAGTTCA |
| Ihh-R | CCGTGTTCTCCTCGTCCTT |
| Dkk1-F | CCATGAACTCAGGTCCATTCT |
| Dkk1-R | ATTCCCTCCCTTCCAATAACT |

**Supplementary Table5. The top 29 significantly enriched signaling pathways with statistical significance**

| **Rank** | **Name** | **pSize** | **NDE** | **pNDE** | **pPERT** | **pG** | **pGFDR** |
| --- | --- | --- | --- | --- | --- | --- | --- |
| \| 1 \| \| --- \| \| 2 \| \| 3 \| \| 4 \| \| 5 \| \| 6 \| \| 7 \| \| 8 \| \| 9 \| \| 10 \| \| 11 \| \| 12 \| \| 13 \| \| 14 \| \| 15 \| \| 16 \| \| 17 \| \| 18 \| \| 19 \| \| 20 \| \| 21 \| \| 22 \| \| 23 \| \| 24 \| \| 25 \| \| 26 \| \| 27 \| \| 28 \| \| 29 \| | \| PI3K-Akt signaling pathway \| \| --- \| \| Rap1 signaling pathway \| \| Protein processing in endoplasmic reticulum \| \| Focal adhesion \| \| MicroRNAs in cancer \| \| ECM-receptor interaction \| \| HIF-1 signaling pathway \| \| Regulation of actin cytoskeleton \| \| Ras signaling pathway \| \| Axon guidance \| \| Gap junction \| \| Amoebiasis \| \| Malaria \| \| Melanoma \| \| Proteoglycans in cancer \| \| Transcriptional misregulation in cancer \| \| Cell cycle \| \| Mineral absorption \| \| Glioma \| \| Wnt signaling pathway \| \| HTLV-I infection \| \| Bladder cancer \| \| Pertussis \| \| Leishmaniasis \| \| Legionellosis \| \| Phosphatidylinositol signaling system \| \| Toll-like receptor signaling pathway \| \| Tuberculosis \| \| Toxoplasmosis \| | \| 351 \| \| --- \| \| 216 \| \| 169 \| \| 206 \| \| 270 \| \| 87 \| \| 111 \| \| 217 \| \| 228 \| \| 129 \| \| 87 \| \| 120 \| \| 48 \| \| 71 \| \| 226 \| \| 179 \| \| 126 \| \| 47 \| \| 65 \| \| 143 \| \| 277 \| \| 38 \| \| 74 \| \| 66 \| \| 58 \| \| 81 \| \| 101 \| \| 177 \| \| 113 \| | \| 36 \| \| --- \| \| 20 \| \| 25 \| \| 25 \| \| 17 \| \| 16 \| \| 14 \| \| 20 \| \| 20 \| \| 14 \| \| 9 \| \| 12 \| \| 6 \| \| 8 \| \| 16 \| \| 12 \| \| 11 \| \| 4 \| \| 7 \| \| 9 \| \| 17 \| \| 4 \| \| 2 \| \| 3 \| \| 6 \| \| 4 \| \| 1 \| \| 8 \| \| 5 \| | \| 0.000000 \| \| --- \| \| 0.000003 \| \| 0.000000 \| \| 0.000000 \| \| 0.000000 \| \| 0.000000 \| \| 0.000003 \| \| 0.000003 \| \| 0.000007 \| \| 0.000015 \| \| 0.000697 \| \| 0.000122 \| \| 0.002045 \| \| 0.000776 \| \| 0.000539 \| \| 0.004239 \| \| 0.000795 \| \| 0.041068 \| \| 0.002134 \| \| 0.018620 \| \| 0.002048 \| \| 0.020623 \| \| 0.612877 \| \| 0.269854 \| \| 0.005342 \| \| 0.188031 \| \| 0.942107 \| \| 0.118216 \| \| 0.205918 \| | \| 0.029985 \| \| --- \| \| 0.011994 \| \| 0.264368 \| \| 0.346327 \| \| 0.423788 \| \| 0.461769 \| \| 0.628186 \| \| 0.762119 \| \| 0.703648 \| \| 0.565217 \| \| 0.016492 \| \| 0.428786 \| \| 0.075462 \| \| 0.245377 \| \| 0.461769 \| \| 0.123938 \| \| 0.920040 \| \| 0.028486 \| \| 0.824588 \| \| 0.107446 \| \| 0.983008 \| \| 0.178411 \| \| 0.006997 \| \| 0.016492 \| \| 0.905547 \| \| 0.031484 \| \| 0.006497 \| \| 0.060470 \| \| 0.039480 \| | \| 0.000001 \| \| --- \| \| 0.000001 \| \| 0.000004 \| \| 0.000005 \| \| 0.000007 \| \| 0.000007 \| \| 0.000023 \| \| 0.000033 \| \| 0.000061 \| \| 0.000106 \| \| 0.000142 \| \| 0.000566 \| \| 0.001509 \| \| 0.001822 \| \| 0.002314 \| \| 0.004492 \| \| 0.006011 \| \| 0.009067 \| \| 0.012919 \| \| 0.014433 \| \| 0.014513 \| \| 0.024302 \| \| 0.027666 \| \| 0.028548 \| \| 0.030625 \| \| 0.036286 \| \| 0.037312 \| \| 0.042468 \| \| 0.047252 \| | \| 0.000045 \| \| --- \| \| 0.000045 \| \| 0.000173 \| \| 0.000173 \| \| 0.000173 \| \| 0.000173 \| \| 0.000468 \| \| 0.000595 \| \| 0.000983 \| \| 0.001528 \| \| 0.001862 \| \| 0.006797 \| \| 0.016710 \| \| 0.018738 \| \| 0.022212 \| \| 0.040431 \| \| 0.050914 \| \| 0.072538 \| \| 0.097916 \| \| 0.099517 \| \| 0.099517 \| \| 0.159067 \| \| 0.171288 \| \| 0.171288 \| \| 0.176402 \| \| 0.198997 \| \| 0.198997 \| \| 0.218408 \| \| 0.234629 \| |
|  |  |  |  |  |  |  |  |

Rank- Rank of which pathway according to total pathway guide score.

Name- Name of pathway.

psize- # of genes in pathway

NDE - # of differentially expressed genes in input list in pathway

pNDE- Raw p value just talking overrepresentation in pathway into account.

pPERT- Raw p-value just taking intermolecular signaling into account.

pG (PGamma)- Raw  p-value taking both overrepresentation and intermolecular signaling into account.

pGFDR- False discovery rate of pGamma.

**Supplementary Table6. IPA upstream regulator analysis**

| Upstream Regulator | Molecule Type | Predicted Activation State | Activation z-score | p-value of overlap | Target molecules in dataset |
| --- | --- | --- | --- | --- | --- |
| TP53 | Transcription regulator | Inhibited | -2.113 | 2.64E-32 | ACTN4,ANTXR1,ANXA3,ANXA8/ANXA8L1,ARL6IP1,AURKB,BCL2L1,BIRC5,BNIP3,CALU,CAMK2B,CCDC80,CCNB1,CCND2,CCNG1,CD97,CDC42EP3,CDC7,CDKN1A,CENPF,CGREF1,CHST12,CLIC4,COL2A1,COL3A1,COL4A1,COPB1,CREB3,CTGF,CTSB,DFFB,DKK1,DOK1,EGFR,EMILIN1,ENPP2,EPS8,EXO1,FAM120A,FBLN2,FHL1,FLRT2,FMO1,FSTL1,GAPDH,GAS1,GMNN,HIF1A,HK2,HSPG2,ID2,IFI16,IGF1R,IKBIP,ITGB5,KIAA0101,KIF23,KITLG,KLHL21,LDHA,LGALS3,Ly6a,MAFB,MBNL2,MCM5,MKI67,MMP13,Mt2,MYH10,NCAPG,NDC80,NDUFA4L2,NID2,NME1,NPNT,OAT,P4HA1,P4HB,PAK3,PDE4B,PDGFA,PDGFRA,PDGFRB,PDIA5,PLA2G16,PLK2,PLOD2,PLTP,PMS2,PPFIBP1,PRELID1,Ptprv,RECQL4,RPN1,RRM1,SEC61A1,SERPINB9,SERPINE2,SERPINH1,SLC2A1,SMC2,SPC25,SPCS2,SREBF1,STMN1,THBS1,THY1,TIMP2,TMEM97,TNFAIP2,TOP2A,TPX2,TSC22D3,TTK,TUBB,TYMS,UGDH,UHRF1,VCAN,VIM,WSB2 |
| XBP1 | transcription regulator | Inhibited | -5.445 | 6.46E-18 | ARCN1,COPB1,COPB2,COPE,COPZ1,CREB3,FKBP10,FKBP11,FKBP14,GOLPH3,GOLPH3L,GORASP2,HSP90B1,HSPA5,IHH,KDELR3,LMAN1,MCFD2,MOGS,PTHLH,RPN1,S100A6,S1PR1,SEC22B,SEC23A,SEC61A1,SPCS2,SRP54,SRPRB,SSR2,SSR3,SURF4,TRAM1,TXNDC5,YIF1A,YIPF5 |
| CD38 | enzyme | Inhibited | -3.448 | 1.42E-15 | ALDOA,ASNS,BNIP3,BNIP3L,CCND2,CHST12,CKAP4,ELL2,EPS8,FAM162A,GALK1,LGALS3,P4HA1,PFKL,PGM1,RAP1GAP,RPN1,S100A6,Scd2,SKA1,SLC16A3,SLC2A1,SPCS2,STK39,THY1,TMEM97,TPI1,TUBB2B,VIM,XBP1 |
| MYC | transcription regulator | Inhibited | -2.54 | 1.16E-14 | ALCAM,ALDOA,Arf2,ARF3,ASNS,ATP13A2,BCAT1,CCNB1,CCND2,CDKN1A,CHST15,CNP,COL2A1,COL3A1,COL4A1,COL8A1,CSPG4,DFFB,EBF1,FAP,FBLN2,FMOD,FRZB,FSTL1,GAPDH,GPC1,HAPLN1,HIF1A,HK2,ID2,IFI16,LDHA,LXN,MCM5,MKI67,MOGS,MRC1,Mt1,Mt2,MTHFR,OAT,PDGFRA,PDGFRB,PFKL,PGAM1,PGK1,RIN3,SERPINE2,SERPINH1,SLC16A3,SLC1A5,SLC2A1,SOX9,SRM,THBS1,THBS2,THY1,TIMP2,TPI1,TXNIP,TYMS |
| IL5 | cytokine | Inhibited | -3.799 | 3.76E-14 | ALDOA,ASNS,BNIP3,BNIP3L,CCND2,CHST12,CKAP4,ELL2,EPS8,FAM162A,GALK1,HSP90B1,HSPA5,P4HA1,PGM1,RAP1GAP,RPN1,S100A6,Scd2,SKA1,SLC16A3,SLC1A5,SLC2A1,SPCS2,STK39,THY1,TMEM97,TPI1,TUBB2B,VIM,XBP1 |
| CSF2 | cytokine | Activated | 3.671 | 3.52E-09 | ANLN,BCL2L1,BIRC5,CCNB1,CDCA2,CDCA5,CDKN1A,CENPE,CHTF18,EXO1,IFITM3,ITGB5,KNTC1,MCM5,MKI67,MRC1,POLE,RECQL4,SKA1,SLC1A5,SMC2,SPC25,STMN1,TLR4,TOP2A,TPX2,UHRF1,XBP1 |
| HIF1A | transcription regulator | Inhibited | -3.798 | 1.15E-08 | ALDOA,AXL,BCL2L1,BNIP3,BNIP3L,CDKN1A,COX4I2,CTGF,CYR61,EGFR,FHL1,GAPDH,HIF1A,HK2,LDHA,MEF2C,P4HA1,P4HA2,PDGFA,PGK1,PLOD2,SDC4,SLC2A1,SLC39A7,SOX9,TCF4,TGFB3,TMEM45A,TNFAIP2 |
| RABL6 | other | Activated | 2.496 | 1.44E-07 | AURKB,CCNB1,CENPF,KIF23,MCM5,NCAPG,NDC80,POLA1,SERPINH1,TMEM97,TOP2A,TPX2,TTK |
| PRL | cytokine | Activated | 3.036 | 2.18E-07 | ANXA3,CCNB1,CDKN1A,COL3A1,CTSB,CTSS,DCN,ECM1,EGFR,LYVE1,Marcks,P4HB,PC,PDGFRA,PDIA5,PDPN,PIM3,TIMP2,VIM |
| NUPR1 | transcription regulator | Inhibited | -4.145 | 8.63E-07 | ADAM22,BNIP3,CASC5,CDCA2,CENPL,CEP97,CKAP2L,COL3A1,CTPS2,CYR61,ELL2,EPHA7,ETV1,EXO1,FAM111A,FAM162A,GAB2,GPR1,HK2,IGF1R,IL13RA1,KIF18A,KIF23,MKI67,MYH10,OSBPL6,P4HA2,POLA2,POLQ,PPFIBP2,RAB38,RPA1,SLC2A1,SPC25,SPIN4,TMPO,TRERF1,UEVLD,XBP1 |
| ARNT | transcription regulator | Inhibited | -2.967 | 1.36E-06 | ALDOA,BNIP3,CCND2,CD81,GAPDH,GAS1,HK2,LDHA,PGK1,S1PR1,SLC2A1,TPI1,VIM |
| CDKN1A | kinase | Inhibited | -3.105 | 2.12E-06 | BIRC5,CCNB1,CDKN1A,CTSB,EDNRA,EXO1,ITGB5,KIAA0101,MBNL2,RRM1,SOX9,STMN1,TOP2A |
| TSC2 | other | Activated | 2.184 | 3.15E-06 | BIRC5,CCND2,CDKN1A,HIF1A,HK2,HSPA5,PDGFRA,PDGFRB,SLC2A1 |
| CTGF | growth factor | Inhibited | -2.023 | 3.41E-06 | Acan,COL2A1,COL8A1,EMILIN1,HAPLN1,HIF1A,KITLG,MBNL2,MMP13,P4HA1,PTH1R,SFRP4 |
| ATF4 | transcription regulator | Inhibited | -2.334 | 3.73E-06 | ASNS,BCAT1,CDKN1A,CHAC1,GARS,GYS1,LGALS3,NID2,Ptprv,SERPINF1,SLC1A5,SLC38A3,SREBF1,WNT11 |
| NKX3-2 | transcription regulator | Inhibited | -2 | 1.70E-05 | COL2A1,FGFR3,IHH,SOX9 |
| mir-223 | microrna | Activated | 2.206 | 2.25E-05 | ALCAM,C5AR1,ID2,IFI16,IGF1R,Ly6a (includes others),MMP13,Mt2,NFIA,RAB38,S100A6,SCARB1,STMN1,TLR4 |
| COMMD1 | transporter | Activated | 2.449 | 3.37E-05 | ALDOA,BNIP3,GRHPR,HK2,LDHA,PGK1 |
| INHA | growth factor | Inhibited | -2.594 | 7.05E-05 | CCND2,COL3A1,COMP,CTGF,CYR61,EGFR,FGFR1,GJC1,ITGA9,KITLG,LMNA,SFRP4 |
| RAB1B | other | Inhibited | -2.588 | 8.57E-05 | ARF4,GORASP2,KDELR3,SEC22B,SERP1,SRP54,YIF1A |
| WT1 | transcription regulator | Inhibited | -2 | 1.06E-04 | BCL2L1,CDKN1A,CLIC4,CSPG4,EPS8,EZR,IGF1R,LGALS3,LMAN1,Scd2,SOX9,VDR |
| TCR | complex | Inhibited | -2.787 | 1.18E-04 | ABLIM1,BCAT1,BCL2L1,CD200,CTSB,ELL2,HIF1A,HSP90B1,HSPA5,NME1,P4HB,PASK,PGK1,PRELID1,SEMA3A,SLC2A1,TLR4,VDR |
| FOXO3 | transcription regulator | Inhibited | -2.311 | 1.25E-04 | BIRC5,BNIP3,CCND2,CDC42EP3,CDKN1A,CTGF,MMP13,Mt1,Mt2,TXNIP,VIM |
| Alpha catenin | group | Inhibited | -2.419 | 1.36E-04 | ADAMTS2,C1QTNF3,CDH11,COL3A1,CTSB,DKK1,FSTL1,MMP13,PDGFRA,THBS2,TNFAIP2,VIM,ZEB2 |
| Hdac | group | Activated | 2.05 | 2.64E-04 | BIRC5,CDKN1A,EDNRA,EGFR,HIF1A,NDRG4,NPNT,PBX1,PGF,TIMELESS,TXNIP,WIF1,ZEB2 |
| FOXO4 | transcription regulator | Inhibited | -2.412 | 4.69E-04 | BNIP3,CDC42EP3,CDKN1A,CTGF,SCD,Scd2 |
| Irgm1 | other | Inhibited | -2.63 | 4.85E-04 | AURKB,CCNB1,ID2,IFI16,MKI67,NCAPG,TK1 |
| SPRY1 | other | Inhibited | -2 | 6.72E-04 | ETV1,FGFR3,ID2,MEST |
| KIAA1524 | other | Inhibited | -2.184 | 6.96E-04 | COL8A1,DCN,FAP,LXN,PDGFRB,PTPRB,SCIN,SERPINE2 |
| PTK2 | kinase | Inhibited | -2.412 | 9.25E-04 | CSPG4,CYR61,P4HA1,PCOLCE2,PDGFRB,THBS1 |
| MYB | transcription regulator | Activated | 2.184 | 9.60E-04 | BCL2L1,BIRC5,CCNB1,KITLG,POLA1,SLC1A5,VIM |
| MGEA5 | enzyme | Inhibited | -2.668 | 1.22E-03 | ABLIM1,ACTN4,BCL2L1,CPE,EFNB2,FGFR1,ITGB5,LAMB1,PLA2G16,S100A6,SERPINB9,SERPINE2,TCF19,THBS1,TIMP2,VIM,WNT11 |
| EP400 | other | Activated | 2.401 | 1.25E-03 | CDKN1A,CENPF,H2AFZ,INCENP,SKA1,UHRF1 |
| E2F6 | transcription regulator | Inhibited | -2.236 | 1.95E-03 | CDC7,EFNA5,GMNN,KIAA0101,LIG1,MCM5,POLA2 |
| BMPR1B | kinase | Inhibited | -2 | 2.01E-03 | Acan,COL2A1,HAPLN1,SOX9 |
| ESRRG | ligand-dependent nuclear receptor | Inhibited | -2.193 | 3.27E-03 | CDKN1A,GAPDH,HK2,LDHA,TPI1 |
| PTGER2 | g-protein coupled receptor | Activated | 2.333 | 4.26E-03 | CENPE,CENPF,CKAP2L,ECM1,MASTL,MKI67,THBS1,TPX2,TTK |
| MAP4K4 | kinase | Activated | 3.138 | 4.34E-03 | ALG9,CHSY1,GRHPR,GYS1,IMPDH1,PAPSS2,PGAM1,PGM1,SCD,SREBF1 |
| MTORC1 | complex | Inhibited | -2.074 | 7.13E-03 | HK2,LDHA,SCD,SLC2A1,SREBF1 |
| ESRRA | ligand-dependent nuclear receptor | Inhibited | -2.401 | 9.72E-03 | FAM102A,GAPDH,HK2,LDHA,Scd2,TPI1,WNT11 |
| WNT5A | cytokine | Inhibited | -2.236 | 9.92E-03 | ENPP2,Ly6a (includes others),MMP13,PGF,TLR4 |
| BMPR1A | kinase | Inhibited | -2 | 1.30E-02 | Acan,COL2A1,HAPLN1,SOX9 |
| SMAD3 | transcription regulator | Activated | 2.114 | 1.38E-02 | ASPN,CCND2,CDKN1A,COL2A1,COL3A1,CTGF,MMP13,S1PR1,TGFB3,VIM |
| PPARG | ligand-dependent nuclear receptor | Inhibited | -2.236 | 1.40E-02 | BIRC5,CDKN1A,HK2,MKI67,NR2F2,PC,PF4,PRKG2,SCARB1,SCD,Slc25a1,SLC2A1,SREBF1,TCF4,TLR4,TSC22D3,TUBB,VDR,VIM |
| RBM5 | other | Activated | 2.186 | 1.47E-02 | IFITM3,MYO1B,RAB1A,SERPINH1,STAT5B |
| VEGFA | growth factor | Activated | 2.229 | 1.76E-02 | CTSB,CTSS,EFNB2,FGFR1,MMP13 |
| EOMES | transcription regulator | Inhibited | -2.213 | 1.92E-02 | CRABP1,PLXNA4,TCF4,TMEM2,TSHZ1 |
| HOXC6 | transcription regulator | Activated | 2 | 2.06E-02 | DCN,FGFR2,PDE4DIP,PDGFRA |
| CD3 | complex | Inhibited | -2.818 | 3.06E-02 | BCL2L1,BIRC5,CCND2,HELLS,HIF1A,HSP90B1,HSPA5,Ly6a (includes others),P4HB,PDE4B,THY1 |
| PPARGC1A | transcription regulator | Inhibited | -2.57 | 3.26E-02 | CDKN1A,COL2A1,HK2,KCNK1,LDHA,PACSIN2,PGAM1,SCD,SLC39A14,SREBF1 |
| IFN alpha/beta | group | Activated | 2.186 | 4.54E-02 | AXL,IFI16,Ly6a (includes others),MRC1,SERPINB9 |
| CNTF | cytokine | Activated | 2 | 4.60E-02 | FGFR1,SCD,SREBF1,VIM |
| HOXD10 | transcription regulator | Inhibited | -2.236 | 4.83E-02 | CAPN6,EZR,NME1,ST8SIA2,TIMP2 |
| PI3K (family) | group | Inhibited | -2.165 | 6.07E-02 | HSPA5,SCARB1,SLC2A1,SREBF1,TXNIP |
| RBPJ | transcription regulator | Inhibited | -2.393 | 6.76E-02 | CDKN1A,GDF10,KITLG,MMP13,PGF,SOX9,TGFB3 |
| LEP | growth factor | Activated | 2.566 | 2.03E-01 | BCL2L1,CDKN1A,CYR61,FGFR1,FGFR2,GAPDH,HSPA5,SCD,Scd2,SREBF1,UGP2,VIM |
| TLR3 | transmembrane receptor | Activated | 2.412 | 1.00E+00 | CPM,IFI16,IL33,Marcks,PLK2,PROCR |
